# Cell-Cycle Dependence of Transcription Dominates Noise in Gene Expression

**DOI:** 10.1371/journal.pcbi.1003161

**Published:** 2013-07-25

**Authors:** C. J. Zopf, Katie Quinn, Joshua Zeidman, Narendra Maheshri

**Affiliations:** Department of Chemical Engineering, Massachusetts Institute of Technology, Cambridge, Massachusetts, United States of America; Brandeis University, United States of America

## Abstract

The large variability in mRNA and protein levels found from both static and dynamic measurements in single cells has been largely attributed to random periods of transcription, often occurring in bursts. The cell cycle has a pronounced global role in affecting transcriptional and translational output, but how this influences transcriptional statistics from noisy promoters is unknown and generally ignored by current stochastic models. Here we show that variable transcription from the synthetic tetO promoter in *S. cerevisiae* is dominated by its dependence on the cell cycle. Real-time measurements of fluorescent protein at high expression levels indicate tetO promoters increase transcription rate ∼2-fold in S/G2/M similar to constitutive genes. At low expression levels, where tetO promoters are thought to generate infrequent bursts of transcription, we observe random pulses of expression restricted to S/G2/M, which are correlated between homologous promoters present in the same cell. The analysis of static, single-cell mRNA measurements at different points along the cell cycle corroborates these findings. Our results demonstrate that highly variable mRNA distributions in yeast are not solely the result of randomly switching between periods of active and inactive gene expression, but instead largely driven by differences in transcriptional activity between G1 and S/G2/M.

## Author Summary

There is an astonishing amount of variation in the number of mRNA and protein molecules generated from particular genes between genetically identical single cells grown in the same environment. Particularly for mRNA, the large variation seen from these “noisy” genes is consistent with the idea of transcriptional bursting where transcription occurs in random, intermittent periods of high activity. There is considerable experimental support for transcriptional bursting, and it is a primary feature of stochastic models of gene expression that account for variation. Still, it has long been recognized that variation, especially in protein levels, can occur because of global differences between genetically identical cells. We show that in budding yeast, mRNA variation is driven to a large extent by differences in the transcriptional activity of a noisy gene between different phases of the cell cycle. These differences are not because of specific cell-cycle regulation, and in some cases transcription appears restricted to certain phases, leading to pulses of mRNA production. These results raise new questions about the origins of transcriptional bursting and how the statistics of gene expression are regulated in a global way by the cell cycle.

## Introduction

At the single-cell level, mRNA and protein levels of regulable genes are often found to be highly variable [1]–[5]. The resulting long-tailed mRNA and protein distributions are well-described by stochastic models [1], [5]–[7] of transcriptional bursting, where a promoter undergoes random and intermittent periods of highly active transcription. Real-time observations of transcription in multiple organisms appear consistent with this behavior [5], [8]–[14]. Thus, both static and dynamic views attribute much of the observed mRNA variability to the stochastic nature of reactions intrinsic to transcription. Consequently, the standard stochastic model of gene expression has been widely used to infer steady-state dynamics [1], [2], [15]–[17].

However, earlier studies examining the origin of variability in protein expression found such variability is not solely due to stochasticity in reactions intrinsic to gene expression, but also extrinsic factors. This was done by looking for correlations in expression between identical copies of one promoter [18]–[20] and/or between that promoter and a global or pathway-specific gene [21], [22]. Not only is the importance of extrinsic factors clear, without time-series measurements the intrinsic noise measured by these techniques may not completely be ascribed to stochastic reactions in gene expression [23], [24]. While global extrinsic factors have been suggested to largely impact translation [1], their influence on transcription and transcriptional bursting is unclear.

The cell cycle has global effects on total protein and RNA synthesis that should play a role in transcription [12], [20], [25]. With few exceptions [20], most deterministic and stochastic models of gene regulation do not account for cell cycle variability. Using both dynamic real-time protein and static single molecule mRNA measurements in single cells, we show that much of the variability in a synthetic tetO promoter typical of noisy genes in yeast is driven by differences in transcription rate between G1 and S/G2/M.

## Results

**Figure 1:**
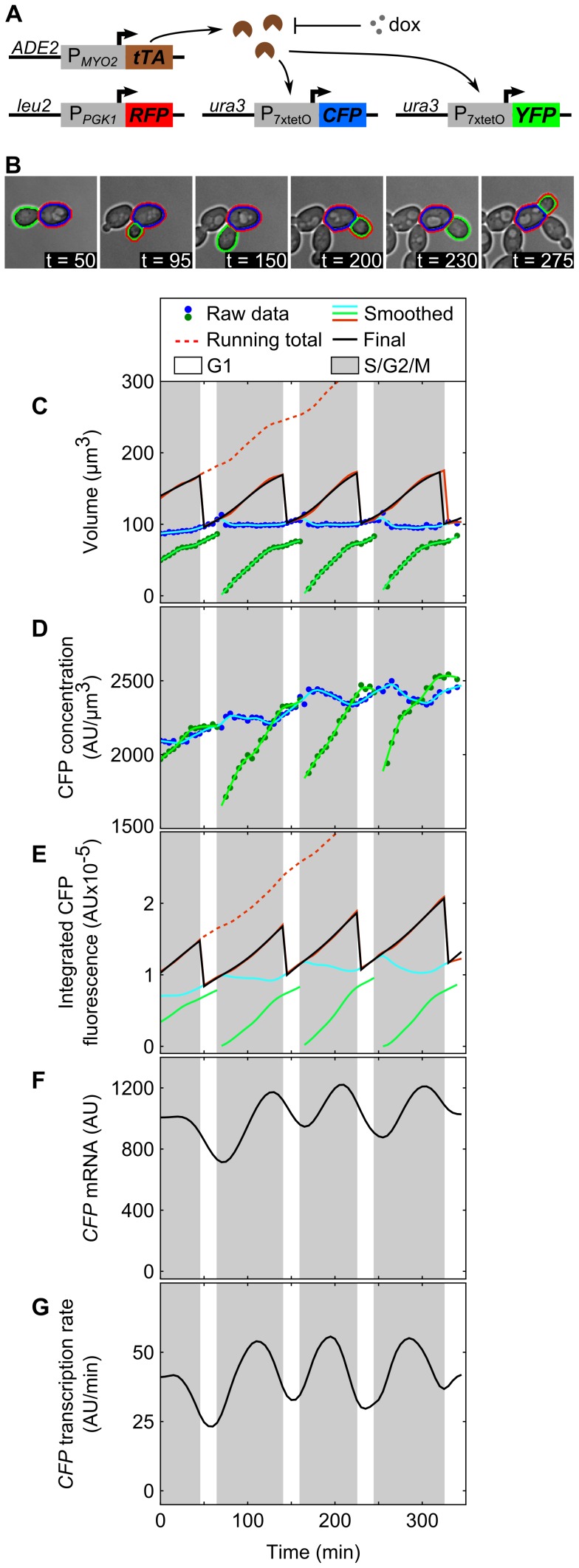
An instantaneous transcription rate is calculated from single-cell fluorescence time series. (A) In the 3-color diploid strain, tet-Trans-Activator (tTA) expressed from P*_MYO2_* activates P_7xtetO_ and is repressed by doxycycline. Homologous copies of P_7xtetO_ drive expression of either *CFP* or *YFP* while P*_PGK1_* drives *RFP* expression constitutively. (B) Monitoring growth of single cells in microfluidic culture. The segmented mother cell, daughter, and contiguous whole cell regions are outlined in blue, green, and red, respectively. For the mother and its daughters, (C) raw volume and (D) CFP concentration time series were smoothed to remove measurement noise; (E) integrated CFP fluorescence was calculated as their product (corresponding regions shown in B). The sum of bud and mother values until division constitutes a whole cell trace, which when extended past division, yields a running total trace that is fit to a differentiable smoothing spline. (F) Calculated relative mRNA levels and (G) instantaneous transcription rate.

We examined cell-cycle dependent effects by microscopically monitoring fluorescent protein expression every 5 minutes in growing monolayers of yeast within a microfluidic chamber. We used a “3-color” diploid yeast strain with homologous 7xtetO promoters (P_7xtetO_) driving either Cerulean (CFP) or Venus (YFP) and a constitutive *PGK1* promoter (P*_PGK1_*) driving tdTomato (RFP) (Fig. 1A). Correlations in transcriptional activity between different promoters allowed distinction between different sources of fluctuations [20], [22]. We developed a method to infer the instantaneous transcription and protein production rates in single cells from the microscopy movies. We segment cells (Fig. 1B), define budding times, track cell lineages and generate time series for volume and protein concentration (measured by average fluorescence, Fig. 1C&D), similar to [26], [27]. Two new features of our analysis enabled us to identify transitions in transcriptional state from these time series: precise assignment of cytokinesis (Fig. S1) and using splines to stably estimate first and second time derivatives (Text S1). The product of volume and protein concentration time series gives total protein, *P*(*t*) (Fig. 1E), from which we infer the protein production rate (proportional to mRNA per cell, *M*(*t*)) (Fig. 1F), and transcription rate, *A*(*t*) (Fig. 1G) using a system of two differential equations describing transcription and translation (Methods). The single-cell, instantaneous transcription rate estimated (Fig. 1G) represents a rate smoothed over a 15–20 minute window (due to measurement and spline fitting errors) and delayed 10–15 minutes due to fluorophore maturation (Text S1).

**Figure 2:**
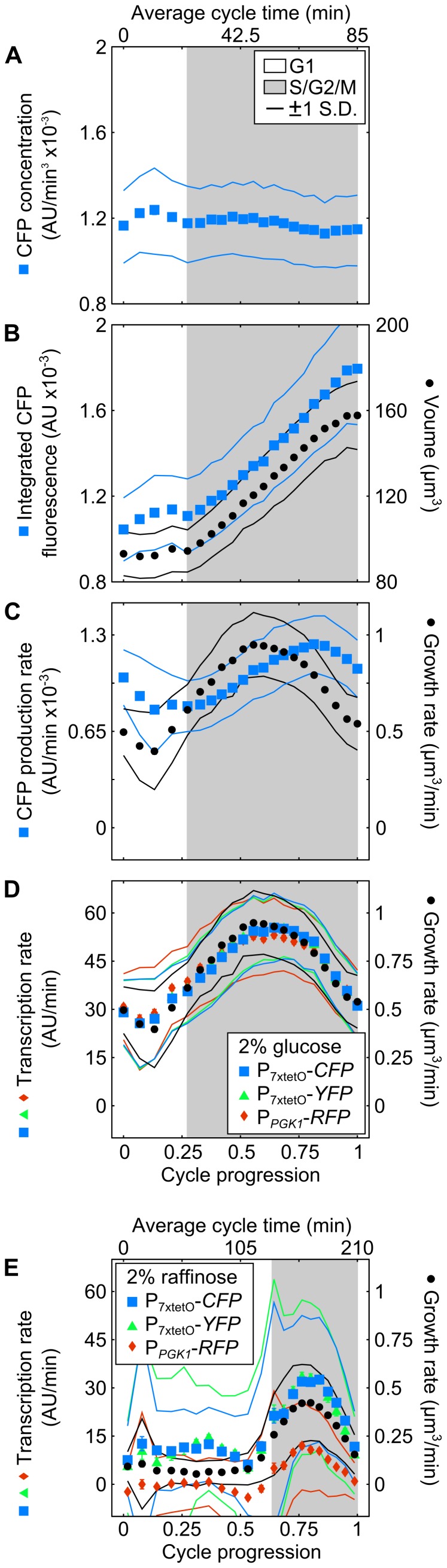
Instantaneous transcription rate in single yeast cells correlates with growth across the cell cycle. Single-cell data was binned by cell-cycle progression and averaged. (A) Because of the small (<10%) decrease in CFP concentration across the cell cycle, (B) total CFP rises rapidly with volume post-bud formation, similar to [27], but at slightly different rates. (C) Mean instantaneous growth and protein production rates are lower in G1 and peak in S/G2/M. Instantaneous transcription rate of P_7xtetO_ and P*_PGK1_* correlates with instantaneous growth rate for growth in (D) 2% glucose (N = 171 cell cycles/bin) and in (E) 2% raffinose (N = 246 cell cycles/bin). *YFP* and *RFP* transcription averages were normalized to that of *CFP* in glucose (D). Error bars represent the bin SEM from bootstrapping. Dotted lines indicate the bin s.d.

To examine the effects of cell-cycle phase on expression, we aligned growth and expression data with respect to cell-cycle progression. We subdivided single-cell time series data between division events, synchronized the data by bud formation time, and rescaled time such that the pre- and post-bud phases mapped to the population average time in those phases. Thus, division occurs at 0 and 1, and all traces bud at the same cell-cycle progression (Fig. S2). While reporter concentration is nearly flat across the cycle (Fig. 2A), cells exhibit a slow growth phase up to bud formation corresponding to G1 and very early S, followed by faster growth in S/G2/M (Fig. 2B), consistent with [27], [28]. The instantaneous protein production rate similarly has two modes, but lags the instantaneous growth rate (Fig. 2C). In contrast, the instantaneous transcription and growth rate correlate, approximately doubling in S/G2 relative to G1 (Fig. 2D). This does not invalidate observations that total protein production rates correlate with average growth rate [29] (Fig. S3). The gradual rise in transcription rate over ∼30 min may mask a sharper change because of smoothing and reporter maturation (Fig. S4). These findings are robust to changes in cells' average growth rate (Fig. 2E and Fig. S5) and remain present even without rescaling time (Fig. S2).

**Figure 3:**
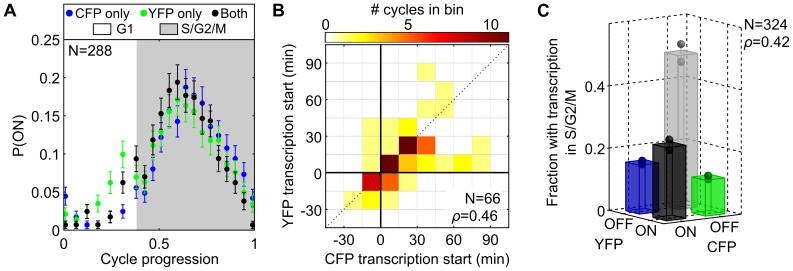
Transcriptional bursts from homologous loci are cell-cycle dependent and partially correlated. The 3-color diploid strain was grown in microfluidics with 50 ng/mL dox, reducing expression from P_7xtetO_. (A) The probability that each P_7xtetO_'s transcription rate is above background (Text S1), computed by averaging binarized individual cell responses (ON = 1, or OFF = 0) in each cell-cycle progression bin, increases after G1. (B) A 2D histogram of activation time for each promoter when both activate (t = 0 at budding). Most activation occurs near budding and is correlated. (C) Classifying all single-cell S/G2/M periods (from (A) plus those following an unobserved G1) by whether each P_7xtetO_ activates reveals correlations in sporadic expression. Error bars represent SEM from bootstrapping.

We next added 50 ng/mL dox to reduce P_7xtetO_ expression in the 3-color diploid to levels where transcription is thought to occur in infrequent, independent bursts at each locus that are presumably resolvable by the real-time analysis. Single-cell traces of transcription rate show occasional “on” periods (Fig. S6) that are restricted to S/G2, generally begin within 20 minutes of bud formation, and last until division (Fig. 3A). If both P_7xtetO_ copies turn on, >70% of the time they do so within 15 minutes of each other (Fig. 3B). The “on” periods are not independent (p<10^−5^, χ^2^ test; ρ = 0.42) at each locus (Fig. 3C). These results are in striking contrast to the view of transcriptional bursting as intrinsically driven with exponential interarrival times [1], [4], [8], [30].

**Figure 4:**
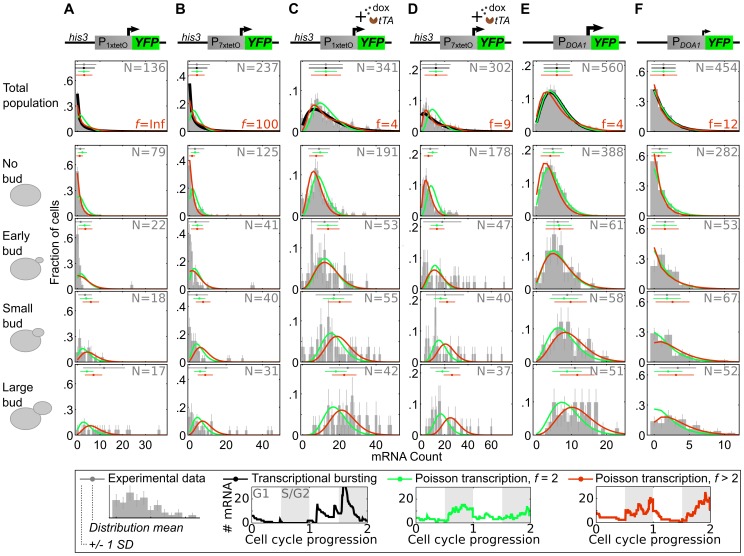
Large differences in transcriptional activity between S/G2/M and G1 depend on promoter. (A) YFP mRNA distributions in a haploid yeast with integrated P_1xtetO_-YFP and no tTA are shown in a column as a function of cell-cycle phase. Horizontal lines above each distribution are the experimental (gray) and predicted mean/standard deviation for different models, with colors shown in the legend at the bottom, calculated by assuming each bud phase represents 1/3 of S/G2/M. (B) As in (A) but with P_7xtetO_. (C&D) As in (A&B) but with tTA and 100 or 500 ng/mL dox added for P_1xtetO_ and P_7xtetO_, respectively. (E) Integrated P*_DOA1_*-YFP with native *DOA1* expressed from a plasmid. Mid log-phase cells were analyzed. (F) As in (E) but late log-phase cells.

We sought further support for these real-time observations by using single-molecule mRNA fluorescence in situ hybridization (FISH) to probe how mRNA numbers in single cells varied with cell-cycle phase, classified based on the presence and size of a bud (Methods). Fig. 4A&B describes mRNA distributions from cells with P_1xtetO_ and P_7xtetO_, but no activator present (basal expression). The G1 distributions are zero-peaked with a long-tail that disappears by early S, suggesting transcription does not occur in G1, consistent with Fig. 3A. Progression through S/G2/M leads to a unimodal non-zero-peaked distribution in G2/M consistent with real-time observations (Fig. 3B) indicating the time when an inactive promoter turns ON in S/G2 is variable. With intermediate expression, there is also increased activity in S/G2/M, but the G1 distribution is qualitatively different: a non-zero peak indicates transcription now occurs in G1 (Fig. 4C&D). However, low transcription activity does not imply G1 inactivity. The weak but constitutive *DOA1* promoter (P*_DOA1_*) [31] has a non-zero G1 peak even with low mRNA levels (Fig. 4E).

While the overall mRNA distributions exhibit excellent fits to a negative binomial predicted by the standard model [1], [32] (Fig. 4), partitioning the data by cell-cycle phase reveals it is incorrect. We sought to understand to what extent the overall variability could be explained by a model with a constant transcription rate that increased by *f*-fold between G1 and S/G2/M (Text S1). When *f = 2* as expected based on S-phase replication, the model qualitatively describes the progression of the observed distributions for P*_DOA1_* (Fig. 4E&F, Table S3, Fig. S7). However, *f>2* better describes tetO promoter measurements, with *f*>100 for basal expression, consistent with no G1 transcription (Fig. 4A–D, Fig. S7). Still, noisy tetO promoters have more variable transcription than this model can generate. The real-time protein data (Fig. S6) also indicate variability in the amount of mRNA produced in S/G2/M. Incorporating this variability by randomizing the timing of the transcription rate transition in the model gives predictions that agree well with P_1/7xtetO_ but not P*_DOA1_* (Fig. S8, Text S1). The mRNA FISH images for tetO promoters tend to have bright spots thought to represent nascent mRNA transcription that are more likely in S/G2/M (Fig. S9) and may indicate “bursty” expression as another source of variability.

**Figure 5:**
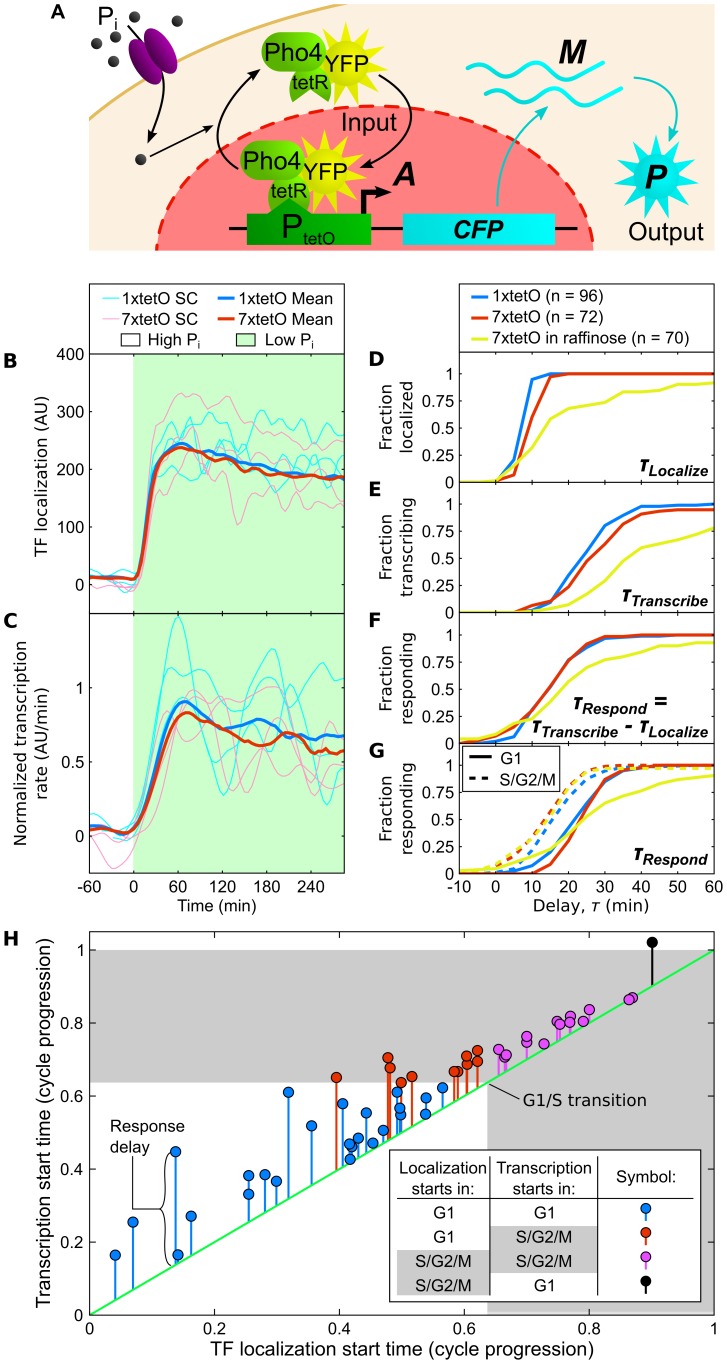
Gene activation depends on cycle phase. P_1xtetO_ and P_7xtetO_ were activated in the kinetic strain background (A) by a step change in phosphate concentration. (B) The time to localization of chimeric Pho4-tetR-YFP and (C) the subsequent kinetic transcription activation are identical for both promoters, when grown in 2% glucose and transcription rate traces are normalized by the average first peak height. Distributions of all single-cell (D) localization and (E) transcription activation delay times were calculated from the time of the phosphate switch to the time to cross an effective threshold (Fig. S11). (F) Each cell's delay to respond is calculated as the difference between the cell's transcription and localization times. (G) Separating the response delay time distributions by cell-cycle phase at the time of localization reveals faster activation post-budding. (H) Long response delays occur only when Pho4-tetR-YFP localizes in G1. Each cell is plotted as a stem where the distance along the abscissa corresponds to the cell-cycle progression at the time of TF localization; the height of the stem endpoint along the ordinate corresponds to the cell-cycle progression at the time of transcription activation; and the length of the stem from the green diagonal to the endpoint represents the response delay. Colors depict phases of localization and activation as in the inset legend. Data shown are for P_7xtetO_ in raffinose.

Finally, we asked if cell-cycle phase affected the kinetics of gene activation by measuring the time to activate P_1xtetO_ and P_7xtetO_ in response to a step change in transcription factor (TF) input. Because TF-regulating pathways in single cells may respond to chemical inducers like dox slowly and at variable times [33], we developed a “kinetic” strain where TF activation was observable in its nuclear localization. The TF Pho4p localizes to the nucleus in response to low phosphate [34]. Reengineering the phosphate pathway (Text S1) allows rapid, reversible control of a Pho4-tetR-YFP fusion capable of activating P_1/7xtetO_-CFP by toggling phosphate concentration (Fig. 5A), with minimal effects on cell growth (Fig. S10).

In response to a step change in phosphate concentration, both TF localization and subsequent gene activation from P_1xtetO_ or P_7xtetO_ driving CFP expression are identical (Fig. 5B&C). The difference between localization and transcription activation timing in single cells (Fig. 5D&E, Text S1) yields a response delay time distribution (Fig. 5F) with a median 17 min delay likely dominated by fluorophore maturation (∼10 min delay, Fig. S12). Therefore, on average both promoters respond quickly to the TF. However, when we separate cells by the cell-cycle phase when TF localization occurs, the post-budding response delay distribution is significantly different from the pre-budding delay distribution (Fig. 5G, 2-sample K-S test: p = 0.05, P_1xtetO_; p<0.001, P_7xtetO_), with median response times in post-budded cells 7 (P_1xtetO_) or 10 (P_7xtetO_) minutes earlier. We repeated the P_7xtetO_ step test in 2% raffinose to extend G1 and better sample G1 cells. Both the median 20 min response delay and the 10 min gap between pre- and post-bud cells' delay (K-S test: p = 0.005) are similar to results in glucose, with prolonged delays in activation restricted to G1 (Fig. 5H, Fig. S13).

## Discussion

Transcriptional bursting, whereby a promoter occasionally transitions from a long-lived inactive to a short-lived active state that produces a burst of mRNA, is commonly invoked to explain the observed single-cell variability in mRNA numbers [1] of noisy genes. In contrast, we demonstrate that large differences in transcriptional activity between G1 and S/G2 that go beyond gene dosage effects drive much of the observed single-cell variability in mRNA numbers. Genome-wide studies in yeast [3], [35] have identified noisy promoters as those associated with strong TATA boxes and highly regulated by chromatin remodeling factors. The tetO promoters, whose core region is derived from the native *CYC1* promoter [36], have similar characteristics. Likewise, constitutive, housekeeping promoters and highly expressed tetO promoters previously associated with low expression variability [2], [3], [31] exhibit only ∼2-fold changes in transcription throughout the cell cycle consistent with gene dosage effects. None of these promoters are classified as cell-cycle regulated [37]. While the cell cycle has been appreciated to be an important source of extrinsic noise, our findings suggest there may be a specific role beyond gene dosage for noisy genes that have not been associated with cell-cycle regulation. Transcriptional bursting may still occur, but it is not needed to explain most of the variability in mRNA levels given the variability in timing of the G1 to S transition [38], [39]. The heretofore unexplored connection between noisy gene expression and large differences in G1 and S/G2 transcriptional activity raises fundamental questions concerning its origin and prevalence amongst noisy genes in various organisms and its implications in stable gene network regulation.

Studies monitoring transcripts in synchronized yeast cells with tiling microarrays identify ∼10% of genes that exhibit cell-cycle-dependent expression [37]. The mechanistic basis of their cycling, through regulation by cyclin-dependent transcription factors, is well-understood. The *CYC1* gene, whose core promoter is present in the tetO promoters, is not part of this class. Because we observed cycle-dependent basal expression for tetO promoters in the absence of any tTA, the *CYC1* gene might be expected to behave similarly. If we surmise that this cycle-dependent expression occurs at a majority of the ∼20% of genes whose expression has been identified in genome-wide studies as noisy [35], why has not previous work, which monitors expression in populations of synchronized cells, identified these genes? First, normalized microarray data represent a mole *fraction* of mRNA species in the population. As such, they will never identify, say, two-fold changes in mRNA *abundance* due to replication when normalized to the concurrent rise in total mRNA [40]. Second, we see large differences in *transcription rate* between G1 and S/G2 but because of the finite mRNA lifetime, differences in mRNA abundance are smaller. The discrepancies are much more pronounced with slower growth conditions (as the mRNA reaches the new steady-state in each growth phase), but the microarray studies were done in fast growth conditions. Third, under conditions with the largest cell-cycle phase-dependent differences in transcription, S/G2 transcription is a *probabilistic* event. Microarray experiments measure the *population average* mRNA mole fraction in each phase, and hence the relative difference between S/G2 and G1 will be quite low on average. Therefore, monitoring expression in single living cells through multiple cell cycles is crucial to observing cell-cycle-dependent transcription.

Single-cell studies do not suffer from the averaging effects of microarray analysis. Still, our data alters the interpretation of static single-cell studies where mRNA/protein distributions are fit to stochastic models of gene expression to infer steady-state dynamics [1], [2], [15], [17]. This difficulty of using static data to pinpoint origins of variability has been anticipated [5], [23] although here we have shown that even static mRNA FISH data can reveal additional dynamic information by disaggregating mRNA distributions by cell-cycle phase. Consistent with our observations, control measurements made in a study examining cycle-dependent mRNA degradation kinetics using mRNA FISH show an approximately 2-fold increase in mRNA levels as cells progress through S/G2/M for the *ADH1* and *DOA1* promoters [25]. Our results suggest that burst sizes and burst frequencies obtained from fitting static mRNA distributions to a negative binomial distribution may be of limited use beyond the fact that they describe the first two moments of the distribution (and hence its noise). If we titrate tTA levels and infer burst statistics from the aggregate distribution, it appears that burst size increases at low expression levels, then burst frequency increases at high expression levels. This transition roughly corresponds to moving from little or no transcription in G1 (with a burst frequency corresponding to one cell cycle) to robust G1 expression (Quinn, Maheshri, unpublished results). Because of the ease of grossly classifying cell-cycle phase in yeast, an exciting and straightforward exercise would be to reanalyze existing mRNA FISH datasets published from multiple labs to determine the prevalence of cycle-dependent expression. Morphological measurements might also be combined with two-color FISH measurements using a second transcript with known cell-cycle regulation [41]. Such an approach with high abundance transcripts has already revealed independent cell and metabolic cycles [42].

Recent real-time single-cell measurements of transcription may not have seen the cycle-dependent transcription we report for various reasons. In the one yeast study, mRNA expression of a housekeeping gene was measured in real-time with no accounting for cell-cycle phase [12]. In mammalian cells [14], transcriptional bursts with a refractory period were inferred from real-time measurements of luciferase protein levels expressed by multiple promoters, but again there was no accounting for cell-cycle phase. The bursts occurred on time scales much shorter than cell-cycle transitions, but revisiting the data still may reveal a cell-cycle phase dependence. Interestingly, real-time measurements of mRNA expression from two housekeeping promoters in undifferentiated single *Dictyostelium* cells do show a weak cell-cycle dependence, with the frequency of transcriptional burst or pulses dropping 2–3 fold from the beginning to the end of the cell cycle [43]. However, *Dictyostelium* lack a G1 period and have an extended G2 [44], [45]; the reduction in expression later in the cell cycle might be due to mitotic repression of transcription, which is a well-established phenomenon in mammalian cells [46]. It remains unclear how widespread cycle-dependent transcription is in other organisms and awaits further study.

What might be the mechanistic basis for the large differences in G1 and S/G2 transcriptional activity? At low expression tetO promoters exhibit transcriptional pulses that originate around the G1 to S transition when DNA replication occurs. S phase progression has been suggested to affect transcription [47] and has been established as important to (de)silence gene loci in a manner that either requires replication fork progression [48], [49] or not [50], [51]. Replication-dependent transcriptional activation of viral genes has been reported in transient transfection assays, and depends on *trans* activators that bind to either the proximal promoter or and enhancer region [52], [53]. In these cases, there does not appear to be cycle-dependent changes in the activity of the *trans* activators. Furthermore, in an *in vitro* system replication potentiates Gal4-VP16 transcriptional activation during S phase [54]. In our case, cycle-dependent activity of tTA can be ruled out because we observe the phenomenon even in the absence of tTA (Fig. 4A&B). In S phase, activity of the Cdc28 kinase leads to upregulation of the basal transcriptional machinery which has been shown to lead to similarly timed pulses of transcription in ∼200 yeast genes not classified as cycle-dependent, but these are enriched in housekeeping genes and *CYC1* did not exhibit this behavior [55]. Therefore, we favor the hypothesis that temporary disruption of a repressed promoter's chromatin architecture during DNA replication could explain the pulse timing (Fig. 3B) and the increased tendency of tetO promoters to not activate until the G1 to S transition even when Pho4-tetR enters the nucleus in G1 (Fig. 5H). (We observe a similar cycle-dependence at the native *PHO5* promoter where gene activation tends to occur in early S/G2 – Zopf, Maheshri, unpublished results.) Because nucleosomes that are deposited following passage of the replication fork consist of an equivalent mixture of maternal and newly synthesized histones [56], as this chromatin matures there may exist a period permissive for transcription. Even if this permissive period is short-lived, once activated, transcription-mediated deposition of histone marks could maintain an active transcription state [57].

The return to inactive state in G1 might be brought about during mitosis. Repressive modifications in general transcriptional machinery, changes in histone marks, and chromatin condensation all contribute to mitotic repression of RNA transcription in higher eukaryotes [58]. In budding yeast, transcription was originally proposed to occur throughout mitosis as total RNA synthesis appeared to increase at and after nuclear migration [59], but recent work shows Cdc14 inhibiting RNA Pol I transcription during anaphase [60]. Still, mitotic repression of RNA Pol II transcription remains unclear, and we did observe an increased number of nascent spots of transcription in cells with large buds indicative of late G2 or M phase (Fig. S9). Exploring genetic and chemical perturbations in the activity of factors involved in chromatin maturation during DNA replication and mitosis for their effect on the cycle-dependent transcription pattern should aid in elucidating precise mechanisms.

Our results should spur the development of new models that incorporate cell-cycle linked pulses of transcription and analyze its effects on the dynamics and function of genetic regulatory networks. Global extrinsic factors influence the behavior [22] and previous work identifies two important roles of cell division: in the stochastic partitioning of cellular contents [24] and in setting the timescale at which extrinsic fluctuations decay [61]. The latter especially has been incorporated in models of global extrinsic fluctuations [62]. Revised models in light of our results may be particularly exciting to develop in networks containing positive feedback loops, where switching from an OFF to ON state relies heavily on the statistics of transcription at low expression. Because here transcription appears prohibited in G1, this would introduce a sizeable refractory period to switching. Finally, since poorer nutrient conditions increase cell division time by extending G1 in yeast, prohibited G1 transcription may alter the dynamics of regulatory networks in cells in different growth conditions in unexpected ways.

## Methods

### Strain and plasmid construction

All *S. cerevisiae* strains were constructed in a W303 background [63] using standard methods of yeast molecular biology [64]. Details of strain and plasmid construction are listed in Tables S1 and S2. The strain referred to as the “3-color” diploid yeast strain contains homologous 7xtetO promoters (P_7xtetO_) driving either Cerulean (CFP) [65] or Venus (YFP) [66] and a constitutive *PGK1* promoter (P*_PGK1_*) driving tdTomato (RFP) [67] (Fig. 1A). Construction of the “kinetic” strain (Fig. 5A) used to measure the rate of gene activation is described in detail in the Text S1.

### Microfluidic culture and time-lapse microscopy

Yeast nitrogen base without phosphate (MP Biomedicals, Santa Ana, CA; #4027-812) was mixed with monobasic potassium phosphate solution (Sigma-Aldrich P8709) to set phosphate levels, and the pH was lowered to 4. Synthetic complete (SC) media contained all amino acids, 2% glucose as the carbon source, and 5000 µM phosphate unless otherwise indicated. For each time series experiment, cells were picked from a single colony freshly grown on a minimal synthetic solid media (no amino acids) with 2% glucose agar, inoculated into SC media, grown overnight on a roller drum at 30°C to OD_600 nm_∼0.1, diluted in fresh media, and grown again for 6–8 hrs to OD_600 nm_∼0.1. These cells were loaded into a pre-washed and Y04C microfluidic plate (CellAsic, Hayward, CA) primed with SC with appropriate carbon source and/or phosphate level, according to the manufacturer's instructions. Cells were perfused with SC at 6 psi throughout the experiment. Flow was controlled using the programmable ONIX system (CellAsic) to rapidly switch between various media conditions discussed in the text. Cell growth and expression was observed using a Zeiss Axio Observer.Z1 inverted microscope at 63× magnification (Zeiss Plan-Apochromat 63×/1.40 Oil DIC). Bright field (BF), BF out of focus (BFOOF, for segmentation) and fluorescence images were acquired every 5 min with a Cascade II EMCCD camera (Photometrics, Tuscon, AZ) using MetaMorph software (Molecular Devices, Sunnyvale, CA), a Lumen 200 metal-halide arc lamp (PRIOR Scientific, Rockland, MA) for fluorescence excitation, appropriate filters for CFP, YFP, and RFP (Chroma Technology Corp, Bellows Falls, VT; set 89006), and acquisition settings optimized for rapid time points. (Detailed protocol available online at  http://openwetware.org/wiki/Maheshri:Internal.)

### Time series analysis

We used custom-written, GUI-based software in MATLAB (Mathworks, Natick, MA) for semi-automated analysis of the fluorescence microscopy movies to extract single-cell volume and fluorescence data series [68]. Briefly, the cell regions were first segmented using a focused and an unfocused bright field image, tracked through time, and assigned mother-bud lineages, all of which were manually curated. Each bud's data were incorporated into its mother's time series until the automatically-assigned cytokinesis time. Next, in order to obtain stable time derivatives for each data series, we implemented a smoothing algorithm based on spline fitting. Finally, the total protein (fluorescence) spline *P*(*t*) for each cell was used to calculate the protein production rate (proportional to mRNA per cell, *M*(*t*)), and the transcription rate, *A*(*t*), using a simple continuous-time model of transcription and translation: 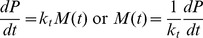
(1)

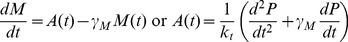
(2)where *γ_M_* is the mRNA degradation rate and *k_t_* is the translation rate of mRNA to protein. We argue translation rate is nearly constant across the cell-cycle (Text S1). Further details of the analytical methods are available in the Text S1.

### Fluorescence in situ hybridization (FISH) to count mRNA in single cells

Single-stranded DNA probes to vYFP were coupled to tetramethylrhodamine (TMR) or indodicarbocyanine (Cy5) fluorophores and probes to tdTomato were coupled to TMR fluorophores, as in [2]. Yeast were grown to mid log-phase (OD_600 nm_ = 0.5–1) then fixed, spheroplasted, hybridized and washed similarly to [69] with modifications as described in [2]. DNA probes at ∼5 µM were diluted 50-fold into hybridization solution containing 10% formamide. Cells were imaged on a Zeiss AxioObserver inverted microscope equipped with a PRIOR Lumen200 mercury arc lamp, a 100×/1.40 objective (Zeiss) and a rhodamine- and Cy5-specific filter set (Chroma Technology Cat. No. 31000v2 and 41024 respectively). For each sample, eight Z-stack images 0.3 microns apart were obtained and analyzed using custom software written in MATLAB based on that used in [2]. The algorithm used to identify spots corresponding to single mRNA applies region-based thresholding and identifies local maxima as spots. Three parameters used by the algorithm can change due to day-to-day variation in staining: (1) the minimum intensity for a pixel to be considered as part of a spot, manually set by examining several z-stacks, picking a threshold that identifies spots and not background, and verified by insuring a false positive rate of <5% in negative control samples; (2) the average intensity of a single mRNA spot, chosen using the mode of spot intensities for lower expressing samples (Fig. S14A), to allow counting of multiple overlapping mRNA; and (3) the threshold intensity at which a spot is classified as a site of nascent transcription, chosen as the transition between the peaked and flat sections of a histogram of spot intensities (∼5–10 fold higher than the intensity of a single spot – Fig. S14A). Mean protein levels in different samples were used as an internal control, and we always verified the ratio of mean protein level to mean mRNA count was consistent across samples and expression levels. Fig. S14B&C are examples of processed images showing mRNA spots.

### Classification of cell-cycle phase in fixed cells

In order to investigate cell-cycle dependence of transcription, we manually classified cells in FISH images in either S/G2/M or G1 based on the presence of a bud. Imaging at low cell density assisted in distinguishing buds from adjacent cells. Cells in S/G2/M were further sub-divided into three equal-sized bins, based on ranked bud size, which approximates progression through the cell cycle (as in Fig. 4).

## Supporting Information

Figure S1 **Division time assignments correlate with nuclear division and cytokinesis time.** At the top, bright field snapshots centered on a single cell are overlaid with RFP fluorescence marking the nucleus. Growth occurs in a microfluidic chamber, and the whole cell volume trace plotted corresponds to the contiguous volume of the central cell in the first image over time. The whole cell volume was obtained as described in the main text and Fig. 1C with automatically assigned budding and division times (beginning and end of gray shaded period, respectively). The cell begins in G1 and grows slowly until bud formation (characteristic of early S phase) at *t* = 50 min. The nucleus migrates to the bud neck in G2 at *t* = 95 min, and divides between mother and daughter during anaphase (A) at *t* = 110 min. The automatically determined division time is *t* = 135 min, at which point the nuclei have separated and the bud neck is narrowed in telophase (T). The subsequent G1 phase begins after the intersection of mother and daughter darkens (indicated by arrow) at the next time point, and by 150 min the dividing cell wall is distinct.
(TIF)

Figure S2 ***In silico*** **synchronization of single-cell time series reveals cell-cycle dependent trends.** Single-cell time series are shown for the 3-color diploid grown in 2% glucose (corresponding to Fig. 2B–D). The first column contains the time series of each cell growing at steady-state. In the second column, each cell-cycle is plotted between division events and synchronized so that budding occurs at *t* = 0 (vertical dashed lines). In the third column, the cell-cycle progression for each cell has been normalized to the average cycle, extending from 0 to 1 with the bud appearing at the average budding point of 0.27. The synchronized single-cell traces demonstrate the average cell-cycle trends depicted in Fig. 2 of the main text are representative of single-cell behavior.
(TIF)

Figure S3 **Instantaneous growth rate and protein production rate vary with doubling time, while volume and protein level depend on nutrient limitation.** (A–C) Data from Fig. 2 in the main text and Figure S5 was averaged over the cell cycle and plotted against the population growth rate (based on average doubling time) in each growth condition. Average instantaneous growth rate correlates fairly well with growth rate as required for balanced growth (A), but average volume depends on the nutrient conditions (B). The average CFP production rate also correlates well with doubling rate but is slightly higher than expected in raffinose (C). This is consistent with observations of total protein production rates correlating with average growth rate [24] since >50% of proteins are diluted through growth [25]. Dividing the average CFP production rate (AU/cell/min) by growth (i.e., dilution) rate yields a prediction for the average total protein per cell at steady state (D).
(TIF)

Figure S4 **Less smoothing results in sharper transitions in average instantaneous growth and transcription rates across cell cycle phases, but general trends are robust to choice of smoothing parameter.** The same raw data in Fig. 2 of the main text and Figure S5 are processed using a more conservative smoothing parameter β = 3 (see Text S1: Determining the smoothing spline parameter) that does not smooth noisy features. After synchronizing and averaging single-cell traces as in Fig. 2, the general trends of the transcription and growth rates are similar for growth in (A) 2% glucose, (B) 2% galactose, (C) 2% raffinose, and (D) low phosphate. Notably, less smoothing leads to sharper transitions at budding and division with roughly constant rates within each cycle phase. Gray areas again show the average S/G2/M phase, and white areas show the average G1 phase.
(TIF)

Figure S5 **Instantaneous transcription rate and growth rate are correlated across the cell cycle in various growth conditions.** Plots were constructed similar to those in Figure 2D&E of the main text for the 3-color strain grown in SC media with 2% galactose as the carbon source (A) or SC with 2% glucose and 100 µM phosphate (B). Error bars represent the bin SEM from bootstrapping. Dotted lines indicate the bin s.d.
(TIF)

Figure S6 **Sporadic transcriptional activation in real-time at two copies of P_7xtetO_.** The 3-color diploid strain was grown with 50 ng/mL doxycycline. Transcription rate time series (solid lines) for four representative cells, one cell in each panel, showing transcription rates from P_7xtetO_ driving either CFP or YFP (blue and green, respectively, top of each panel). Both promoters are off in G1, may or may not turn ON in S/G2 (with a transcription rate that exceeds the dotted black line denoting the detection limit). In contrast, a constitutively expressed RFP (red) from P*_PGK1_* (bottom of each panel) is always ON and exhibits the usual cell-cycle dependent fluctuations. Colored, dashed lines show the corresponding relative mRNA level resulting from transcriptional activity. Vertical dashed lines demarcate cell divisions.
(TIF)

Figure S7 **Summary of each model's fit to S/G2/M-specific mRNA distributions.** (A–F) Strains as in Fig. 4, but comprising a specific comparison of the aggregate S/G2/M distribution and its mean and standard deviation based on the models presented in Fig. 4 and Figure S8. Gray bars and horizontal lines represent the experimental S/G2/M distribution and its mean and standard deviation.
(TIF)

Figure S8 **mRNA distributions from P_1/7xetO_ are better fit by introducing variable timing in the transition from G1 to increased S/G2/M transcription rates.** (A–F) As in Fig. 4, but with the model modified to incorporate a random, uniformly distributed transition from G1 to S/G2/M transition rates occurring during a 40 min window after budding.
(TIF)

Figure S9 **Frequency of bright nascent mRNA spots increase in S/G2/M for tetO promoters.** The fraction of cells with a nascent spot in G1 and through S/G2/M is shown for expression from P_1xtetO_ and P_7xtetO_ at (A&B) basal and (C&D) intermediate expression levels. Strains are identical to those in Fig. 4A–D. (E&F) Fraction of both RFP and YFP nascent spots in a 2-color P_1xtetO_ diploid, where red, green and yellow correspond to the percentage of cells with RFP spots only, YFP spots only, and both spots. While the fraction of cells with nascent spots is larger in (E), their average intensity is less than half the intensity of those in (F), consistent with the lower expression observed in (E). The correlation in the presence of nascent spots from each promoter suggests some degree of coordinated expression. No nascent spots were observed in expression from the constitutive P*_DOA1_*.
(TIF)

Figure S10 **Growth rate decreases gradually after phosphate removal in kinetic experiments.** During step tests on the kinetic strain, the average instantaneous growth rate decreases below normal rates ∼100 minutes after the switch to low external phosphate. Measurements of the activation delay distribution are restricted to this period. The initial rise is due to most cells in the observed population beginning bud growth quasi-synchronously.
(TIF)

Figure S11 **Determining transcription activation and effective TF nuclear localization times in single cells during step tests.** After the switch to low phosphate at *t* = 0 (gray shaded area), a sample cell begins to express CFP (A, cyan) in response to the TF localizing to the nucleus (C, green). The inferred CFP transcription rate (B, blue) rises after the TF localizes to an effective level. Transcription and localization transition times for each cell are then found in 5 calculation steps (corresponding to the red numbers on plots). (**1**) The transcription threshold (horizontal black dashed line, B) is set at 50% of the steady-state transcription rate in the population, determined at a time well after the step as discussed in Methods. This threshold point corresponds well to the beginning of a rise in observable protein (A). (**2**) Because of the 10–15 min lag in observing transcription (*τ*_obs_) due to protein maturation, the TF localization level 15 min prior to the observed time of transcription activation is noted. (**3**) Steps 1 and 2 were repeated for all cell traces creating a distribution of localization values (inset in C). (**4**) The effective TF localization threshold is set at the 5^th^ localization percentile (vertical, dashed line in inset). This corresponds to a TF level where many cells do activate transcription suggesting that nuclear TF levels are no longer limiting. Any resulting delays in transcription activation are interpreted as due to the transcription process and not due to a lack of nuclear TF. (**5**) Each cell's localization time is then determined when nuclear TF levels cross the effective TF localization threshold (horizontal red dashed line, C).
(TIF)

Figure S12 **Measurement of maturation rate for each fluorescent protein.** The 3-color strain was grown in microfluidics in synthetic media. After three hours, the chamber was perfused with the same media now containing 30 µg/mL cycloheximide to inhibit translation. The average intensity in each cell continued to increase for each fluorescent reporter, representing fluorophore maturation from the immature protein pool. (A) Single-cell, raw concentration time series were fit (black line) to Eqn. (S3) (*γ_P_* fixed at 0 for RFP) and (B) the histograms of the measured maturation half-lives ln(2)/*k_m_* are shown. The medians are 10 min, 32 min, and 150 min for CFP (top panel), YFP (middle), and RFP, respectively.
(TIF)

Figure S13 **Transcription response to a step change in TF is slower in G1.** Single-cell TF localization and CFP transcription rate time series are shown for the kinetic step tests in Fig. 4 of the main text. Despite nearly identical localization profiles at the single cell level and on average, the transcription response is slower for cells in which localization occurred in G1 compared to those in which localization occurred after budding (number of cells in G1 (n_G1_) or S/G2/M (n_S/G2/M_) for each experiment: P_1xtetO_, n_G1_ = 20, n_S/G2/M_ = 76; P_7xtetO_, n_G1_ = 23, n_S/G2/M_ = 49; P_7xtetO_ in raffinose, n_G1_ = 53, n_S/G2/M_ = 17).
(TIF)

Figure S14 **mRNA FISH image analysis.** (A) Histogram of the mean pixel intensity of spots detected as mRNA in a population of cells with green, blue and red lines showing the parameters selected to analyze the spots. The threshold (green line) is the minimum pixel intensity for a pixel to be considered as a spot, selected to keep false positives <4% in a negative control sample without the fluorescent reporter and be consistent with manual visual inspection of a subset of images. Multiple mRNAs that overlap in the z-projection appear as a brighter spots. The mode of the histogram (blue line) is selected as the intensity of a single mRNA and spots that are > = 2-fold brighter are counted as multiple spots by normalizing with the mode threshold. Very bright spots (>4-fold brighter than a single mRNA spot in the flat region of the histogram – red line) are thought to be formed by sites of nascent transcription if they align with the nucleus and are automatically identified as those with intensities in the flat region of the histogram (red line). (B) Images of cells with mRNA counts measured by mRNA FISH. *(top)* Bright-field overlaid with blue DAPI-stained nucleus and *(middle)* the maximum projection of eight images fluorescent rhodamine staining within a Z-stack. *(bottom)* mRNA and nascent transcription sites identified by the spot-counting algorithm are marked with a red or magenta dot respectively. Nascent spots align with the blue DAPI-stained nucleus. (C) Micrographs of cells from three of the samples in (B) with *(top)* classification of cell-cycle stage as G1 (yellow), early-S/G2 (orange) or later S/G2/M (red) and *(bottom)* the maximum projection of eight images fluorescent rhodamine staining within a Z-stack.
(TIF)

Figure S15 **Simulation of realistic total protein time series to determine optimal smoothing parameter for spline fit.** Using the method described in Text S1, noise characteristics of experimental integrated fluorescence data before splining (A, representative single cell traces from the P_1xtetO_ and P_7xtetO_ step tests) were used to simulate baseline protein time series (B). (C–E) *A*(*t*) was simulated for a step up at *t* = 0 from zero to an oscillating steady state with the period of a typical cell cycle (*E, t_cyc_* = 100 min, P∶T = 2∶1). A simulated mRNA (D, *μ* = 10) and total protein trace (*C*) were calculated from *A*(*t*) using Eqns. (S5–8), with noise added to the protein time series *ξ*(*t*) similar to the high-frequency, measurement noise observed in data (*σ* ^2^ = 2.4e4). A smoothing spline was fit to the simulated protein time series and the corresponding mRNA and transcription rate time series were inferred using Eqns. (1–2) in the Methods. (F) The simulation was repeated for all combinations of parameters described in the text that span observed characteristics of protein time series across all experiments. For each parameter set (panels), 100 noisy, asynchronous traces were generated and the residual error between the simulated and the inferred transcription was found across a range of smoothing parameters β (colored lines). The chosen β = 300 (vertical, dashed line) minimizes the residual error across all parameter sets and was used for all data sets. (G) Sample comparisons of simulated and inferred transcription rate *μA*(*t*) (using β = 300) for each parameter set with *t_cyc_* = 100 min. Black dotted line represents the half the steady state average for *t*>0 (the threshold for determining activation in a step test).
(TIF)

Figure S16
**mRNA levels fall exponentially with the expected degradation rate after periods of strong transcription.** For the single cell in the bottom panel of Figure S6, the CFP transcription rate (blue) and mRNA level (purple) show sporadic transcription at low expression levels. At the end of a strong “on” period (vertical, dashed line), where transcription rate drops below the threshold (horizontal, dotted line), the inferred mRNA level drop is consistent with the expected first-order degradation process 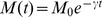 (red line), where *γ* is the mRNA degradation rate and *M_0_* is a constant.(TIF)

Figure S17 **Translational capacity is not cell-cycle dependent.** The average protein to mRNA ratio of cells grouped by cell-cycle phase (based on bud size) is shown for P_1/7xtetO_ and P*_DOA1_*. This ratio exhibits no clear cell-cycle dependent trend, indicating that cell-cycle dependent changes in translation rate are not a dominant source of changes in protein level across the cell-cycle. Error bars are s.d. obtained by bootstrapping.
(TIF)

Figure S18 **Simulations to estimate the time accuracy of the transcription rate transition time assignments.** (A) Transcription rate time series (thin colored lines) are inferred for noisy protein data simulated from the square pulse transcription rate generator function (thick, blue line) as in Figure S15 (n = 1000). Here a protein maturation step is included in simulating the protein data, but not in the transcription rate inference. (B) Activation and (D) deactivation times “observed” for each inferred transcription trace in *A* are delayed 10–15 min relative to the true times (blue histogram compared to vertical, red line) with a mean absolute deviation of ∼2 min. (C) Allowing the mRNA degradation rate γ_M_ in Eqn. (S6) to vary during simulation, but inferring transcription rate using the single, average γ_M_ value, results in greater dispersion in the sample traces. The “observed” activation times did not vary much more, but deactivation time mean absolute deviation increased to ∼8.4 min (black histograms in B and D).
(TIF)

Table S1 **Yeast strains used in this study.**
(PDF)

Table S2 **Plasmids used in strain construction.**
(PDF)

Table S3 **Comparing experimental mRNA expression distributions to simple models.**
(PDF)

Text S1 **Supplementary methods and discussion.**
(DOCX)
