# Supplementary figures and images for: Cell-Cycle Dependence of Transcription Dominates Noise in Gene Expression
Source: PLoS Comput Biol. 2013 Jul 25;9(7):e1003161. doi: 10.1371/journal.pcbi.1003161 (PMC3723585; doi:10.1371/journal.pcbi.1003161)

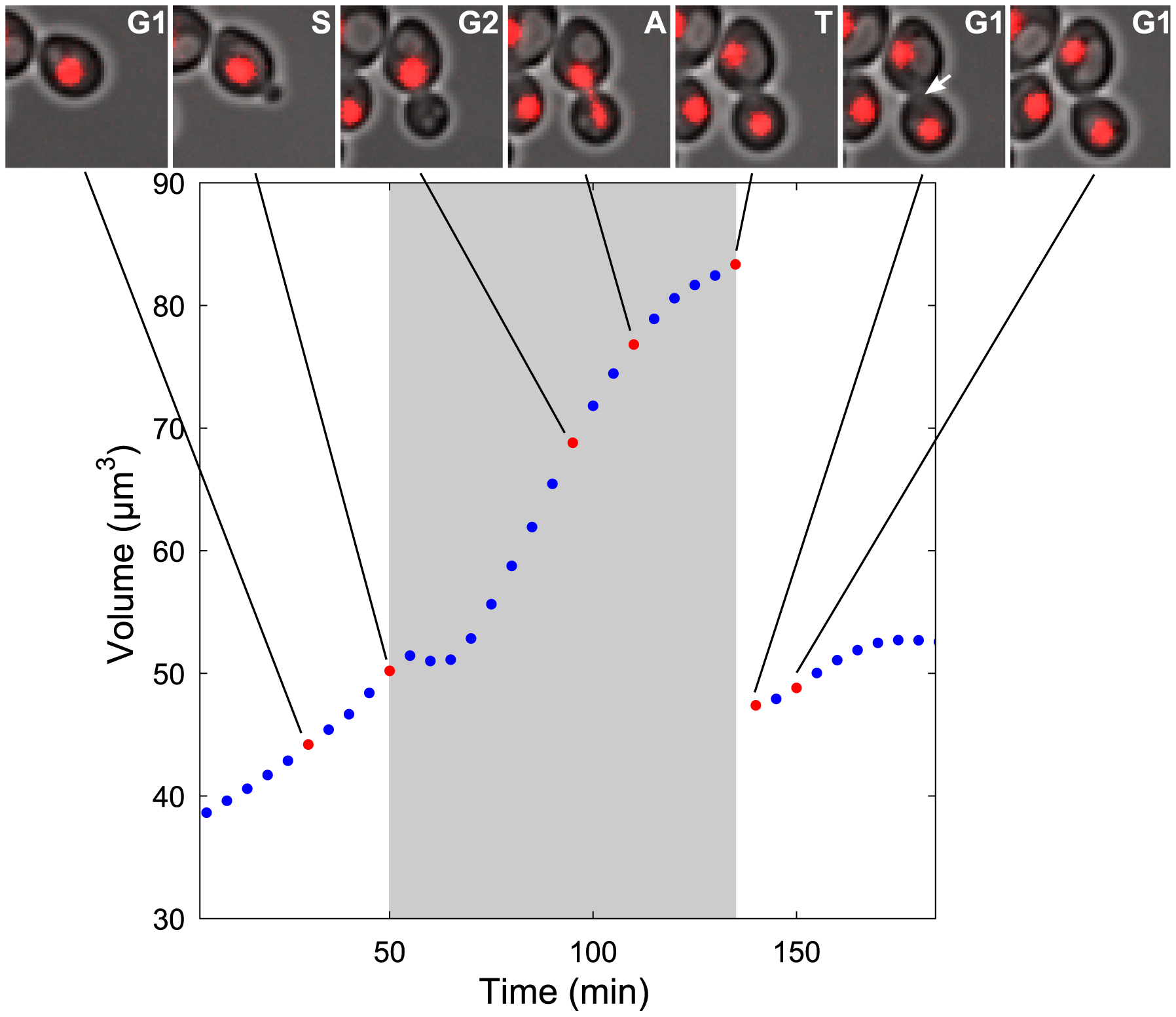

Supplement: Figure S1 — Division time assignments correlate with nuclear division and cytokinesis time. At the top, bright field snapshots centered on a single cell are overlaid with RFP fluorescence marking the nucleus. Growth occurs in a microfluidic chamber, and the whole cell volume trace plotted corresponds to the contiguous volume of the central cell in the first image over time. The whole cell volume was obtained as described in the main text and Fig. 1C with automatically assigned budding and division times (beginning and end of gray shaded period, respectively). The cell begins in G1 and grows slowly until bud formation (characteristic of early S phase) at t = 50 min. The nucleus migrates to the bud neck in G2 at t = 95 min, and divides between mother and daughter during anaphase (A) at t = 110 min. The automatically determined division time is t = 135 min, at which point the nuclei have separated and the bud neck is narrowed in telophase (T). The subsequent G1 phase begins after the intersection of mother and daughter darkens (indicated by arrow) at the next time point, and by 150 min the dividing cell wall is distinct. (TIF) [file pcbi.1003161.s001.tif]

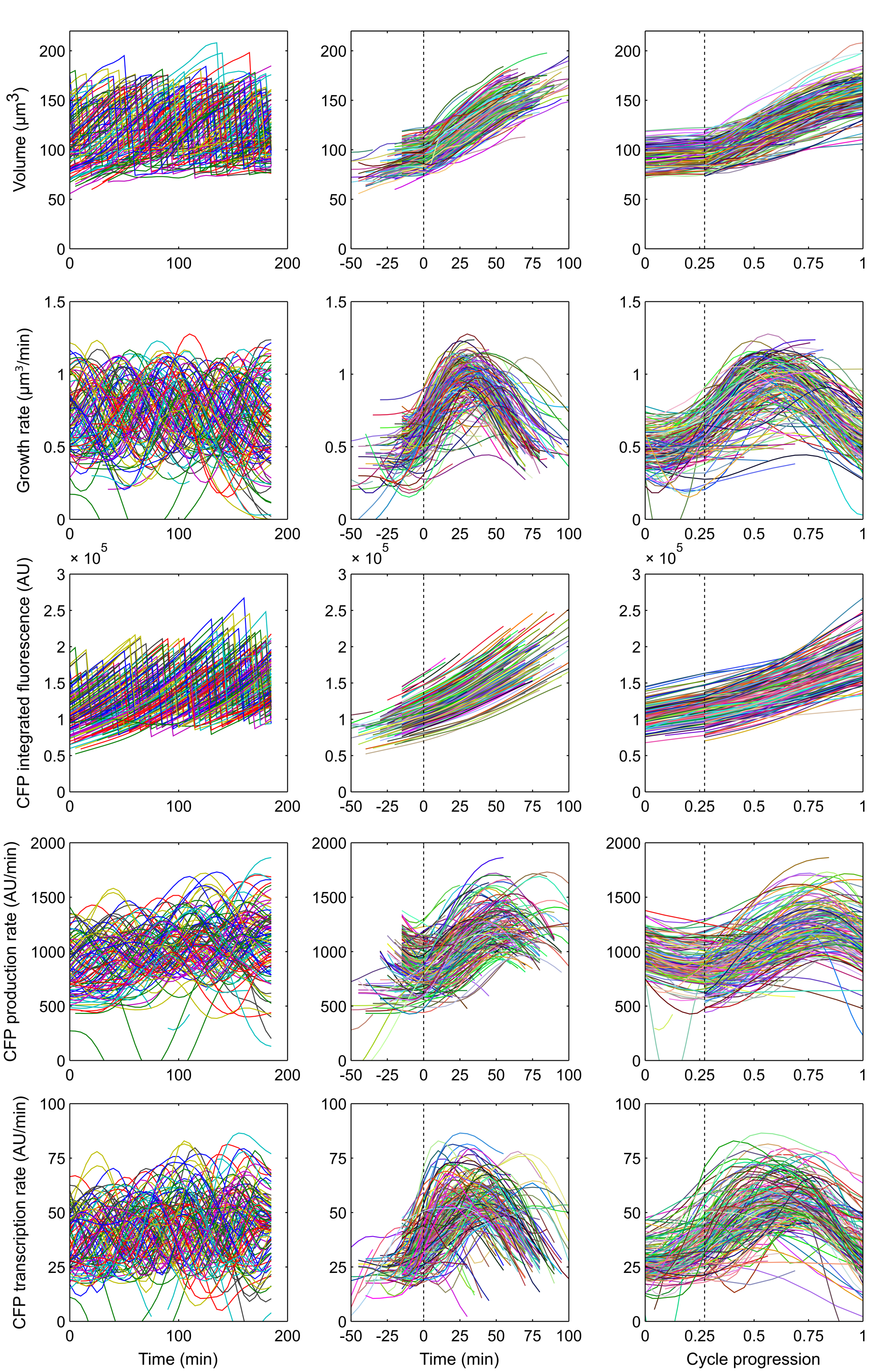

Supplement: Figure S2 — In silico synchronization of single-cell time series reveals cell-cycle dependent trends. Single-cell time series are shown for the 3-color diploid grown in 2% glucose (corresponding to Fig. 2B–D). The first column contains the time series of each cell growing at steady-state. In the second column, each cell-cycle is plotted between division events and synchronized so that budding occurs at t = 0 (vertical dashed lines). In the third column, the cell-cycle progression for each cell has been normalized to the average cycle, extending from 0 to 1 with the bud appearing at the average budding point of 0.27. The synchronized single-cell traces demonstrate the average cell-cycle trends depicted in Fig. 2 of the main text are representative of single-cell behavior. (TIF) [file pcbi.1003161.s002.tif]

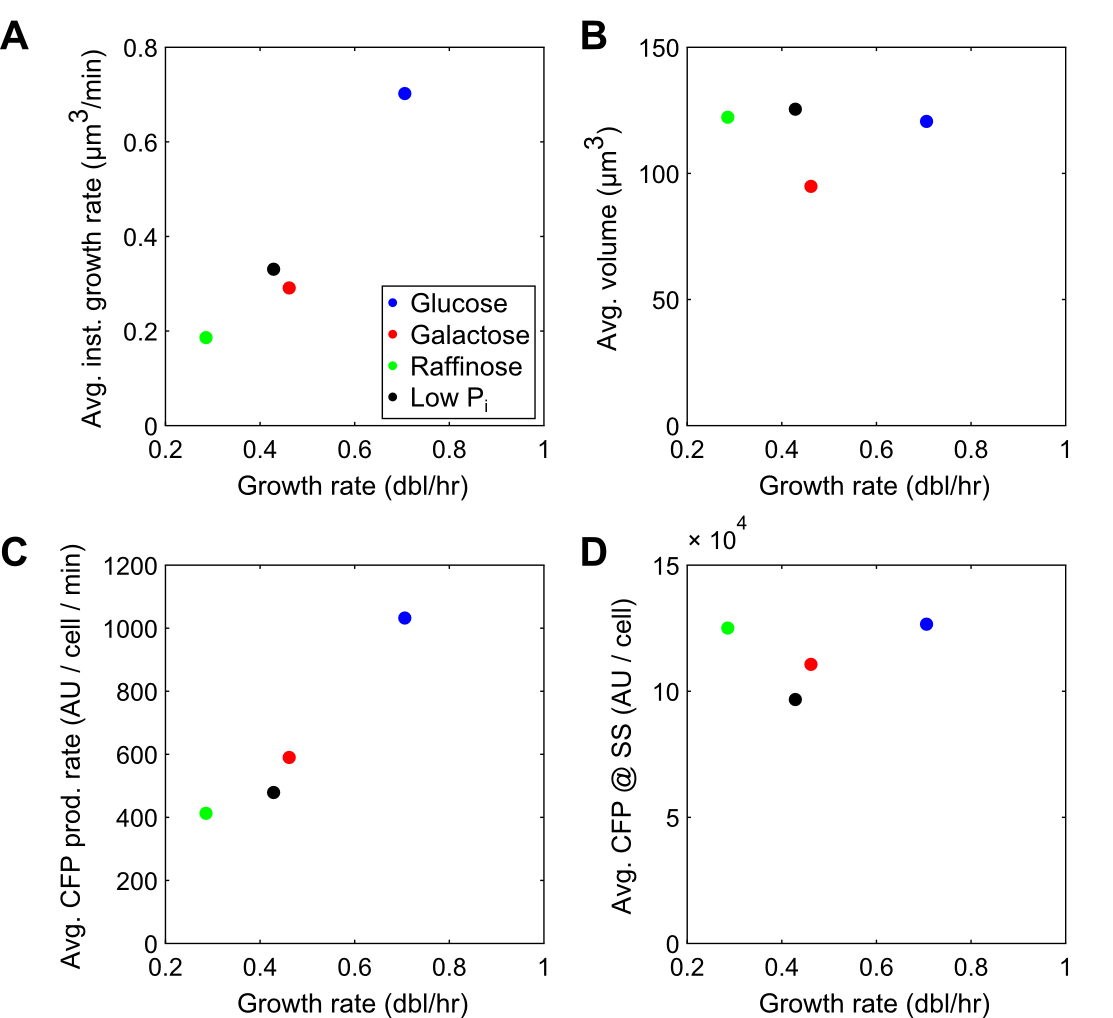

Supplement: Figure S3 — Instantaneous growth rate and protein production rate vary with doubling time, while volume and protein level depend on nutrient limitation. (A–C) Data from Fig. 2 in the main text and Figure S5 was averaged over the cell cycle and plotted against the population growth rate (based on average doubling time) in each growth condition. Average instantaneous growth rate correlates fairly well with growth rate as required for balanced growth (A), but average volume depends on the nutrient conditions (B). The average CFP production rate also correlates well with doubling rate but is slightly higher than expected in raffinose (C). This is consistent with observations of total protein production rates correlating with average growth rate [24] since >50% of proteins are diluted through growth [25]. Dividing the average CFP production rate (AU/cell/min) by growth (i.e., dilution) rate yields a prediction for the average total protein per cell at steady state (D). (TIF) [file pcbi.1003161.s003.tif]

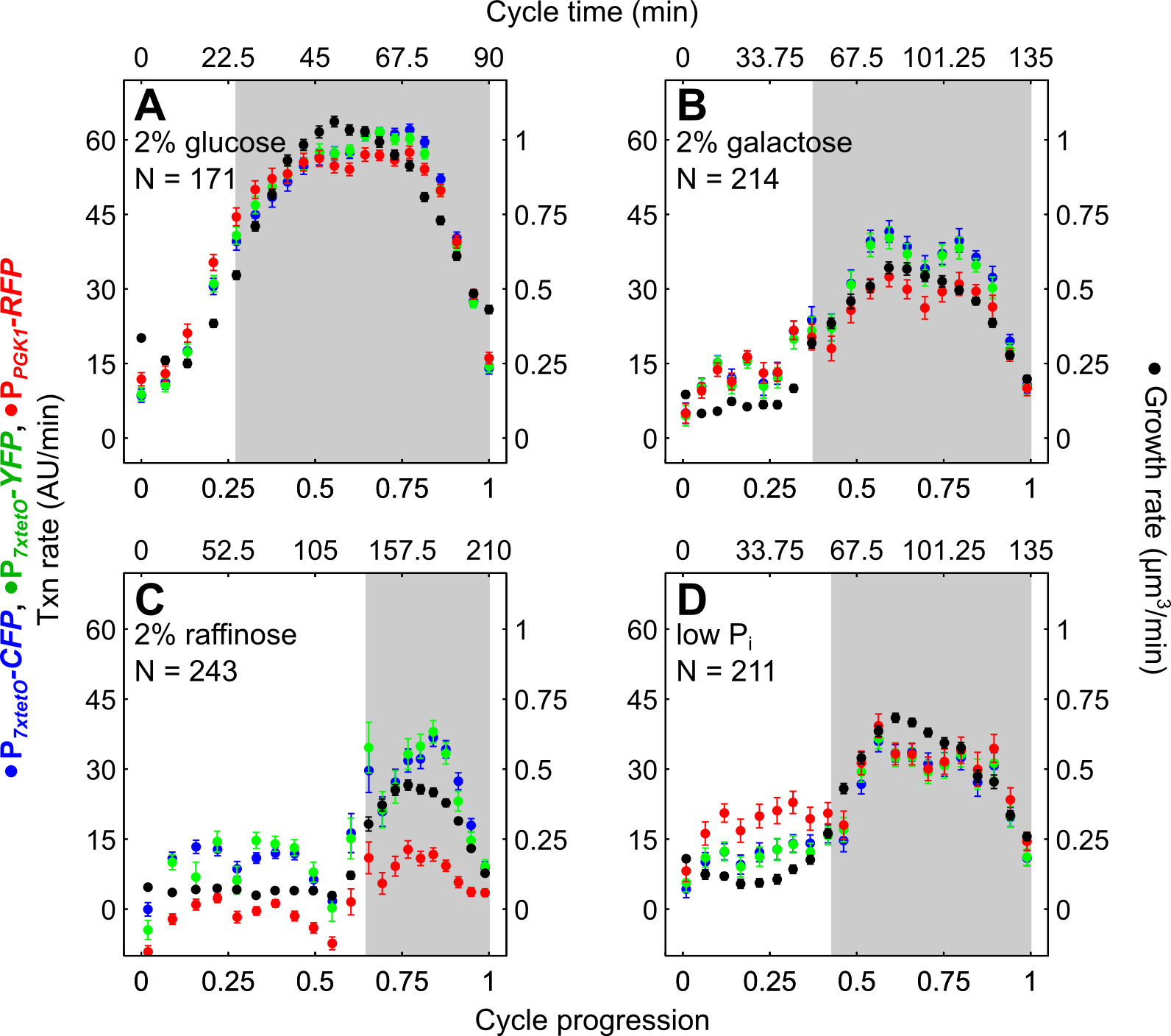

Supplement: Figure S4 — Less smoothing results in sharper transitions in average instantaneous growth and transcription rates across cell cycle phases, but general trends are robust to choice of smoothing parameter. The same raw data in Fig. 2 of the main text and Figure S5 are processed using a more conservative smoothing parameter β = 3 (see Text S1: Determining the smoothing spline parameter) that does not smooth noisy features. After synchronizing and averaging single-cell traces as in Fig. 2, the general trends of the transcription and growth rates are similar for growth in (A) 2% glucose, (B) 2% galactose, (C) 2% raffinose, and (D) low phosphate. Notably, less smoothing leads to sharper transitions at budding and division with roughly constant rates within each cycle phase. Gray areas again show the average S/G2/M phase, and white areas show the average G1 phase. (TIF) [file pcbi.1003161.s004.tif]

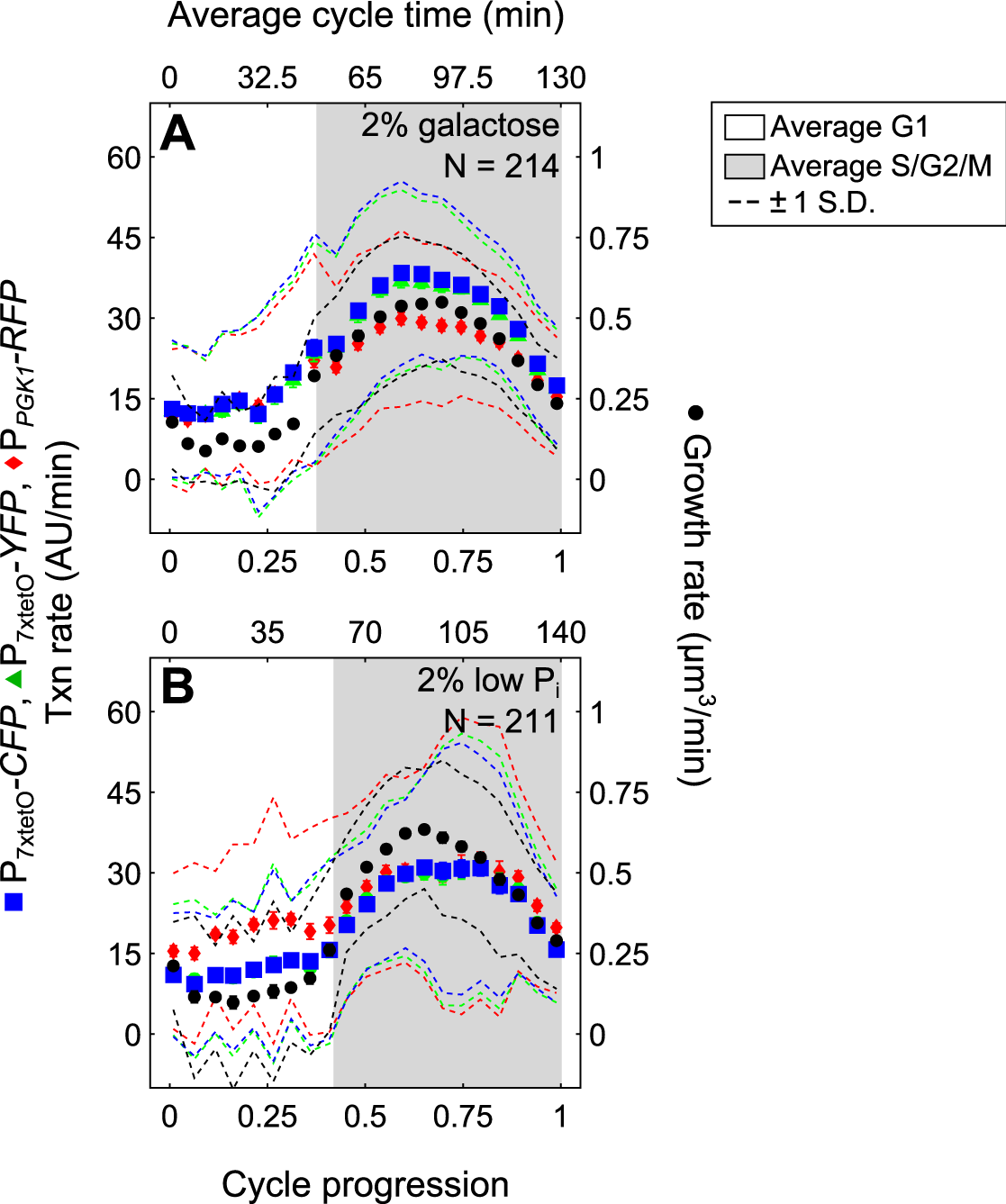

Supplement: Figure S5 — Instantaneous transcription rate and growth rate are correlated across the cell cycle in various growth conditions. Plots were constructed similar to those in Figure 2D&E of the main text for the 3-color strain grown in SC media with 2% galactose as the carbon source (A) or SC with 2% glucose and 100 µM phosphate (B). Error bars represent the bin SEM from bootstrapping. Dotted lines indicate the bin s.d. (TIF) [file pcbi.1003161.s005.tif]

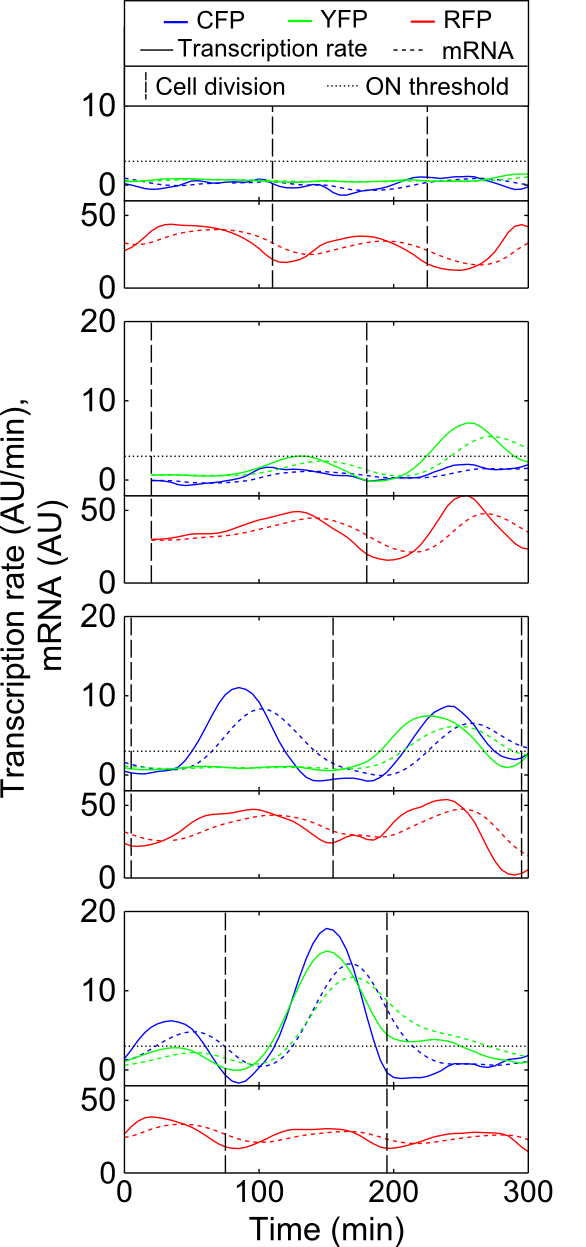

Supplement: Figure S6 — Sporadic transcriptional activation in real-time at two copies of P7xtetO. The 3-color diploid strain was grown with 50 ng/mL doxycycline. Transcription rate time series (solid lines) for four representative cells, one cell in each panel, showing transcription rates from P7xtetO driving either CFP or YFP (blue and green, respectively, top of each panel). Both promoters are off in G1, may or may not turn ON in S/G2 (with a transcription rate that exceeds the dotted black line denoting the detection limit). In contrast, a constitutively expressed RFP (red) from PPGK1 (bottom of each panel) is always ON and exhibits the usual cell-cycle dependent fluctuations. Colored, dashed lines show the corresponding relative mRNA level resulting from transcriptional activity. Vertical dashed lines demarcate cell divisions. (TIF) [file pcbi.1003161.s006.tif]

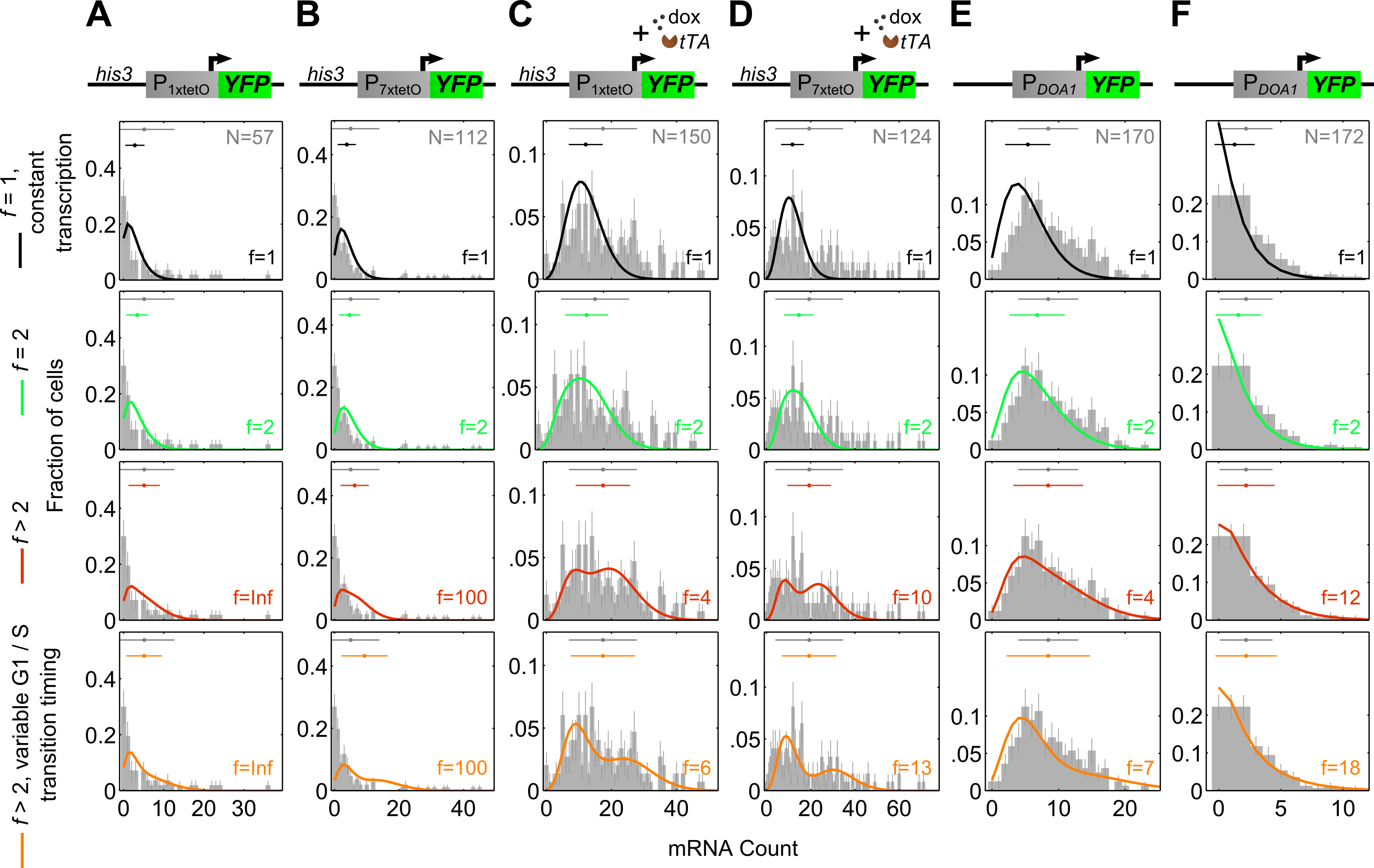

Supplement: Figure S7 — Summary of each model's fit to S/G2/M-specific mRNA distributions. (A–F) Strains as in Fig. 4, but comprising a specific comparison of the aggregate S/G2/M distribution and its mean and standard deviation based on the models presented in Fig. 4 and Figure S8. Gray bars and horizontal lines represent the experimental S/G2/M distribution and its mean and standard deviation. (TIF) [file pcbi.1003161.s007.tif]

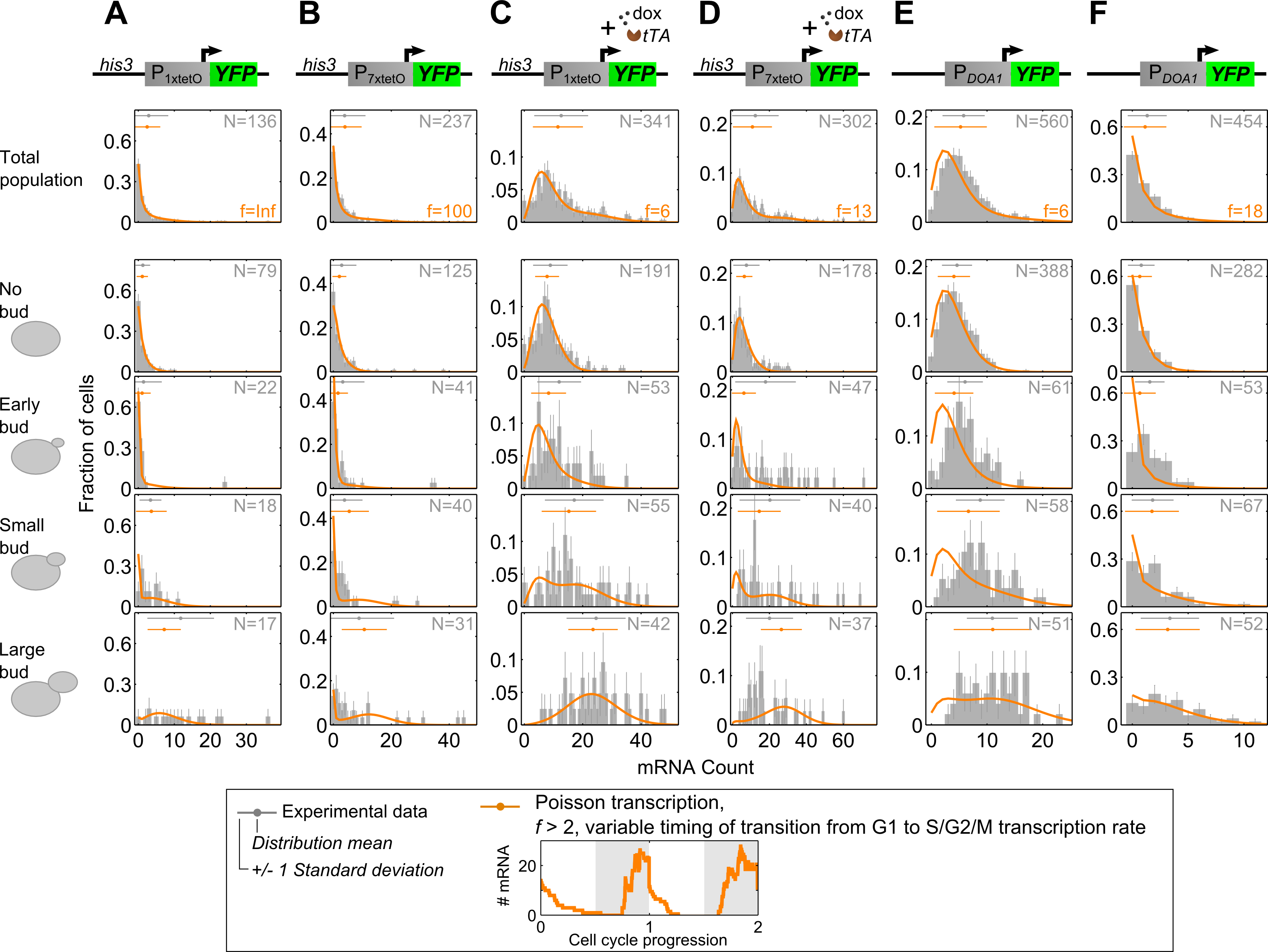

Supplement: Figure S8 — mRNA distributions from P1/7xetO are better fit by introducing variable timing in the transition from G1 to increased S/G2/M transcription rates. (A–F) As in Fig. 4, but with the model modified to incorporate a random, uniformly distributed transition from G1 to S/G2/M transition rates occurring during a 40 min window after budding. (TIF) [file pcbi.1003161.s008.tif]

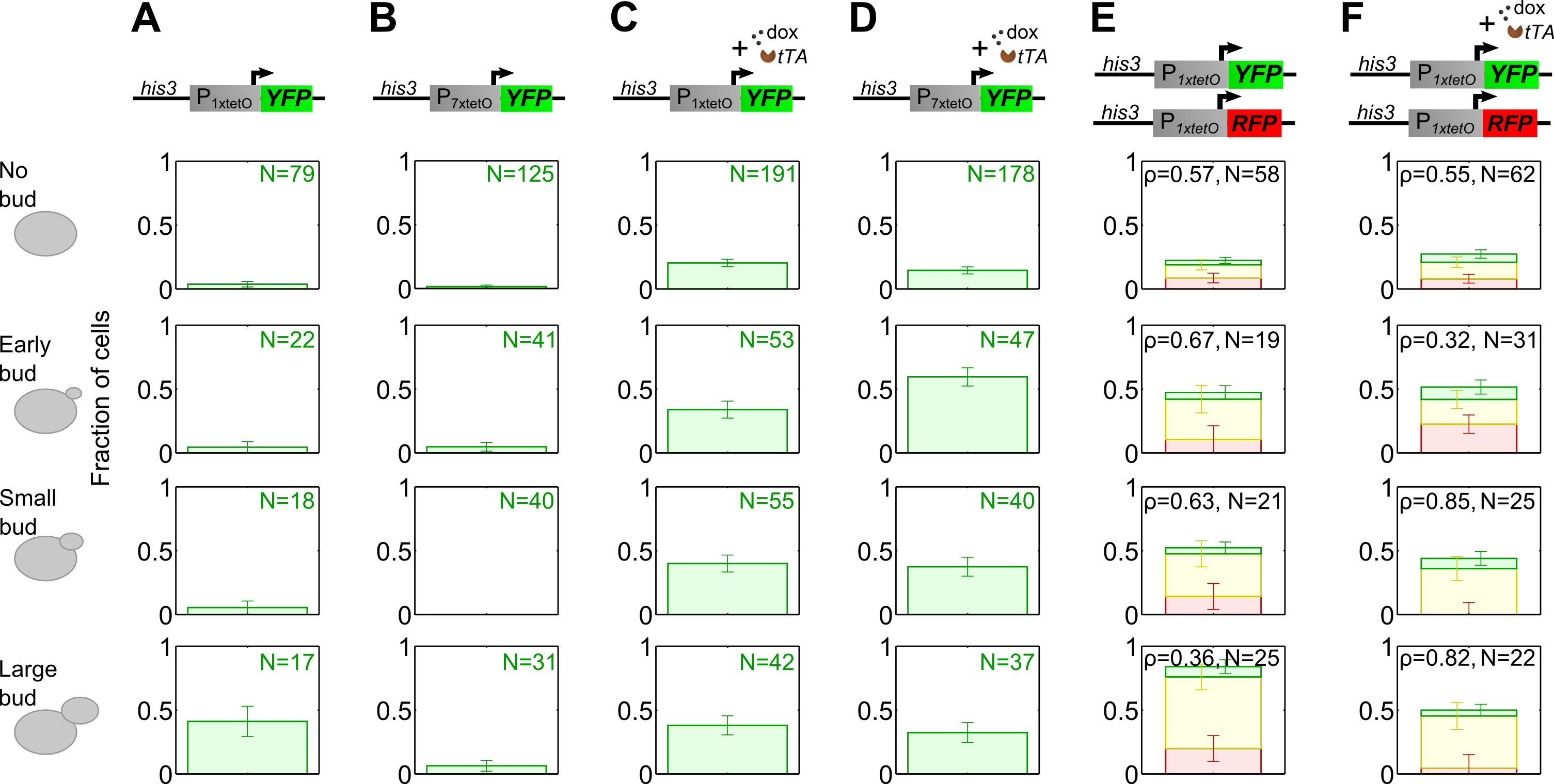

Supplement: Figure S9 — Frequency of bright nascent mRNA spots increase in S/G2/M for tetO promoters. The fraction of cells with a nascent spot in G1 and through S/G2/M is shown for expression from P1xtetO and P7xtetO at (A&B) basal and (C&D) intermediate expression levels. Strains are identical to those in Fig. 4A–D. (E&F) Fraction of both RFP and YFP nascent spots in a 2-color P1xtetO diploid, where red, green and yellow correspond to the percentage of cells with RFP spots only, YFP spots only, and both spots. While the fraction of cells with nascent spots is larger in (E), their average intensity is less than half the intensity of those in (F), consistent with the lower expression observed in (E). The correlation in the presence of nascent spots from each promoter suggests some degree of coordinated expression. No nascent spots were observed in expression from the constitutive PDOA1. (TIF) [file pcbi.1003161.s009.tif]

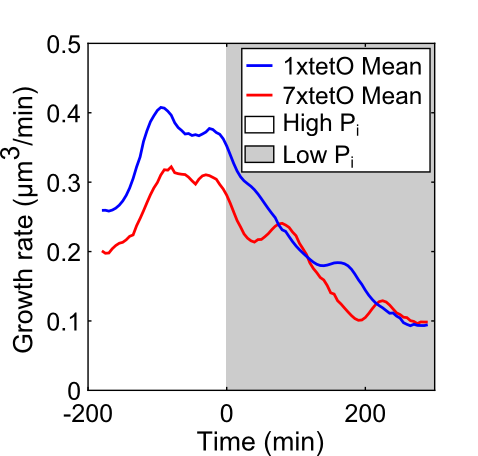

Supplement: Figure S10 — Growth rate decreases gradually after phosphate removal in kinetic experiments. During step tests on the kinetic strain, the average instantaneous growth rate decreases below normal rates ∼100 minutes after the switch to low external phosphate. Measurements of the activation delay distribution are restricted to this period. The initial rise is due to most cells in the observed population beginning bud growth quasi-synchronously. (TIF) [file pcbi.1003161.s010.tif]

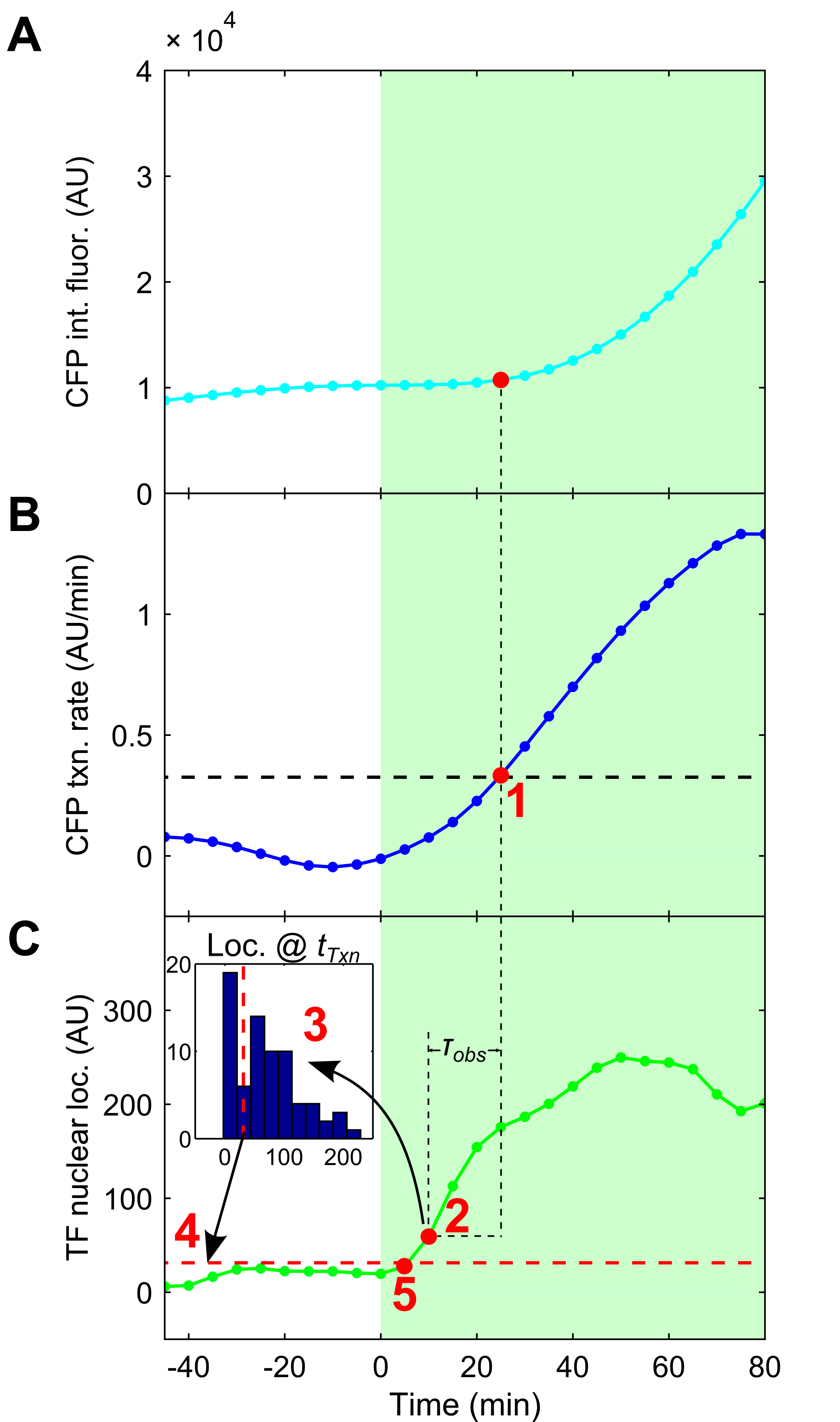

Supplement: Figure S11 — Determining transcription activation and effective TF nuclear localization times in single cells during step tests. After the switch to low phosphate at t = 0 (gray shaded area), a sample cell begins to express CFP (A, cyan) in response to the TF localizing to the nucleus (C, green). The inferred CFP transcription rate (B, blue) rises after the TF localizes to an effective level. Transcription and localization transition times for each cell are then found in 5 calculation steps (corresponding to the red numbers on plots). (1) The transcription threshold (horizontal black dashed line, B) is set at 50% of the steady-state transcription rate in the population, determined at a time well after the step as discussed in Methods. This threshold point corresponds well to the beginning of a rise in observable protein (A). (2) Because of the 10–15 min lag in observing transcription (τ obs) due to protein maturation, the TF localization level 15 min prior to the observed time of transcription activation is noted. (3) Steps 1 and 2 were repeated for all cell traces creating a distribution of localization values (inset in C). (4) The effective TF localization threshold is set at the 5th localization percentile (vertical, dashed line in inset). This corresponds to a TF level where many cells do activate transcription suggesting that nuclear TF levels are no longer limiting. Any resulting delays in transcription activation are interpreted as due to the transcription process and not due to a lack of nuclear TF. (5) Each cell's localization time is then determined when nuclear TF levels cross the effective TF localization threshold (horizontal red dashed line, C). (TIF) [file pcbi.1003161.s011.tif]

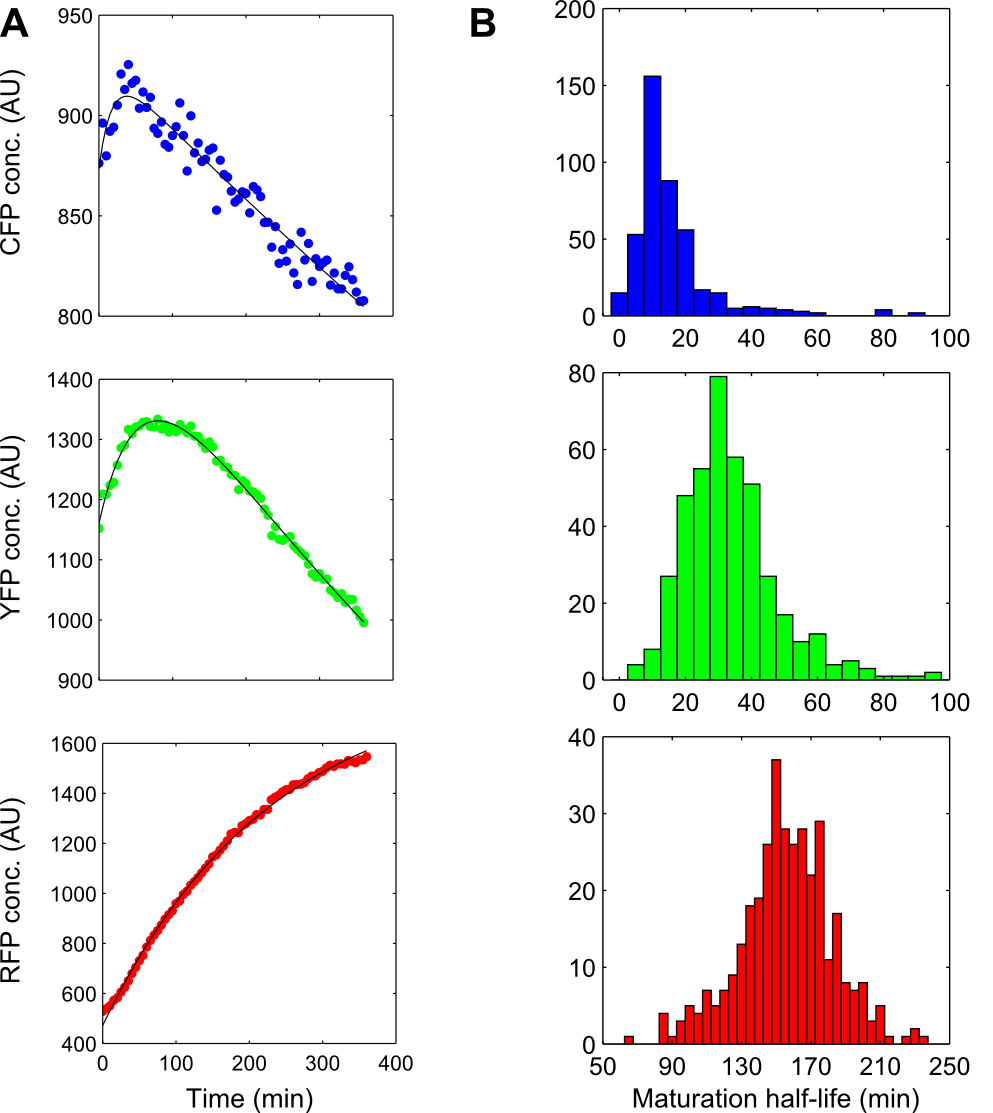

Supplement: Figure S12 — Measurement of maturation rate for each fluorescent protein. The 3-color strain was grown in microfluidics in synthetic media. After three hours, the chamber was perfused with the same media now containing 30 µg/mL cycloheximide to inhibit translation. The average intensity in each cell continued to increase for each fluorescent reporter, representing fluorophore maturation from the immature protein pool. (A) Single-cell, raw concentration time series were fit (black line) to Eqn. (S3) (γP fixed at 0 for RFP) and (B) the histograms of the measured maturation half-lives ln(2)/km are shown. The medians are 10 min, 32 min, and 150 min for CFP (top panel), YFP (middle), and RFP, respectively. (TIF) [file pcbi.1003161.s012.tif]

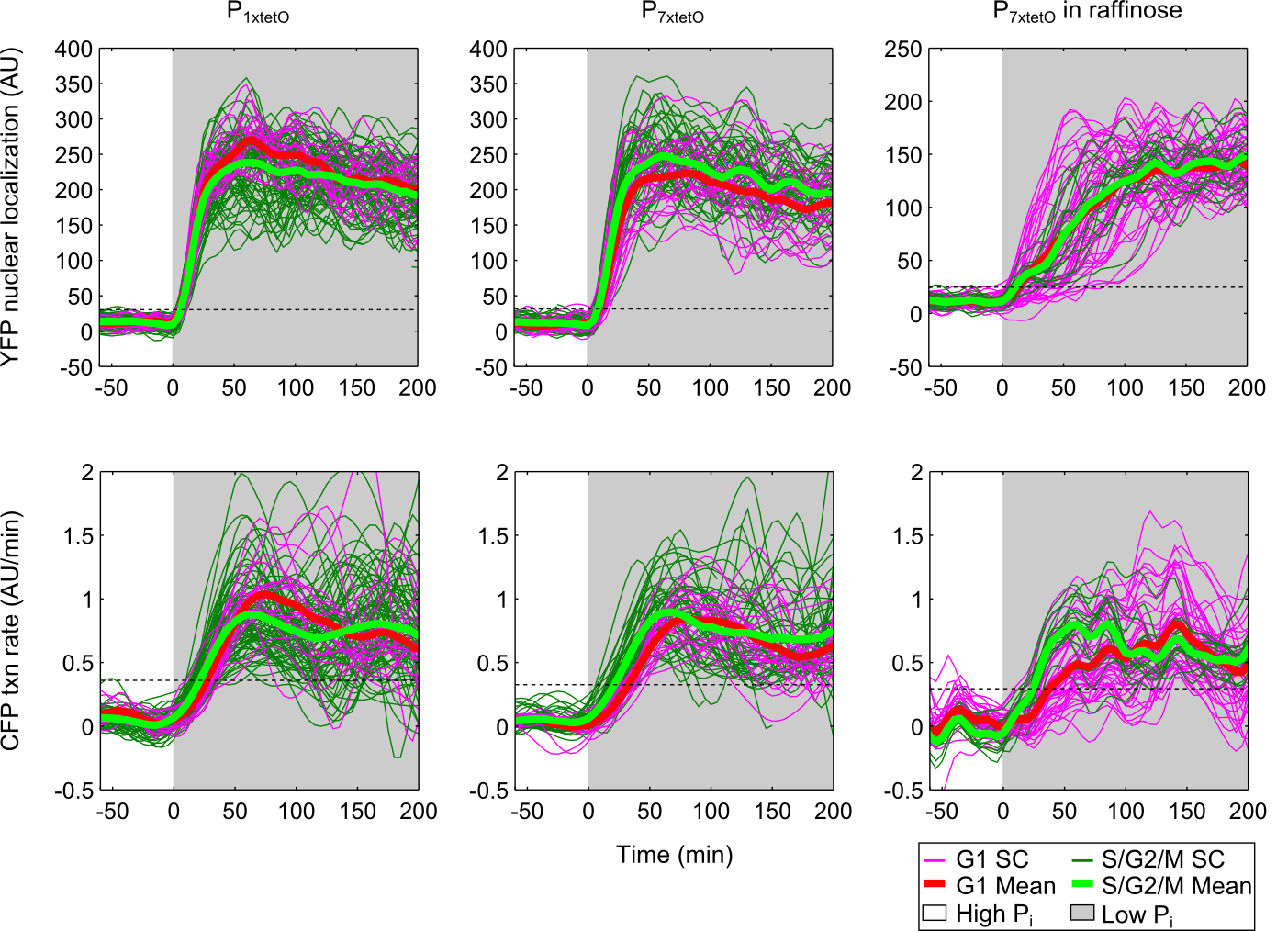

Supplement: Figure S13 — Transcription response to a step change in TF is slower in G1. Single-cell TF localization and CFP transcription rate time series are shown for the kinetic step tests in Fig. 4 of the main text. Despite nearly identical localization profiles at the single cell level and on average, the transcription response is slower for cells in which localization occurred in G1 compared to those in which localization occurred after budding (number of cells in G1 (nG1) or S/G2/M (nS/G2/M) for each experiment: P1xtetO, nG1 = 20, nS/G2/M = 76; P7xtetO, nG1 = 23, nS/G2/M = 49; P7xtetO in raffinose, nG1 = 53, nS/G2/M = 17). (TIF) [file pcbi.1003161.s013.tif]

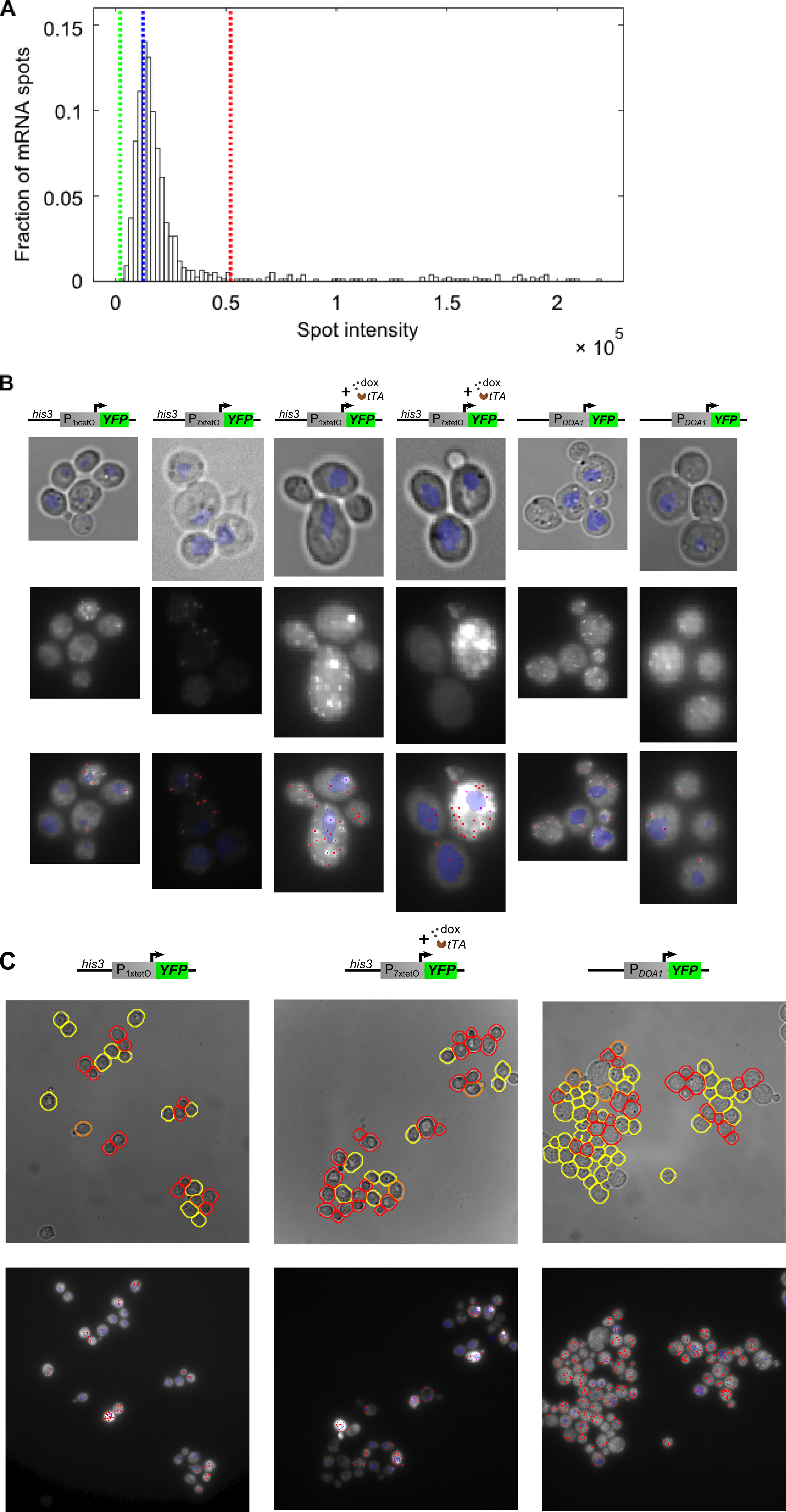

Supplement: Figure S14 — mRNA FISH image analysis. (A) Histogram of the mean pixel intensity of spots detected as mRNA in a population of cells with green, blue and red lines showing the parameters selected to analyze the spots. The threshold (green line) is the minimum pixel intensity for a pixel to be considered as a spot, selected to keep false positives <4% in a negative control sample without the fluorescent reporter and be consistent with manual visual inspection of a subset of images. Multiple mRNAs that overlap in the z-projection appear as a brighter spots. The mode of the histogram (blue line) is selected as the intensity of a single mRNA and spots that are > = 2-fold brighter are counted as multiple spots by normalizing with the mode threshold. Very bright spots (>4-fold brighter than a single mRNA spot in the flat region of the histogram – red line) are thought to be formed by sites of nascent transcription if they align with the nucleus and are automatically identified as those with intensities in the flat region of the histogram (red line). (B) Images of cells with mRNA counts measured by mRNA FISH. (top) Bright-field overlaid with blue DAPI-stained nucleus and (middle) the maximum projection of eight images fluorescent rhodamine staining within a Z-stack. (bottom) mRNA and nascent transcription sites identified by the spot-counting algorithm are marked with a red or magenta dot respectively. Nascent spots align with the blue DAPI-stained nucleus. (C) Micrographs of cells from three of the samples in (B) with (top) classification of cell-cycle stage as G1 (yellow), early-S/G2 (orange) or later S/G2/M (red) and (bottom) the maximum projection of eight images fluorescent rhodamine staining within a Z-stack. (TIF) [file pcbi.1003161.s014.tif]

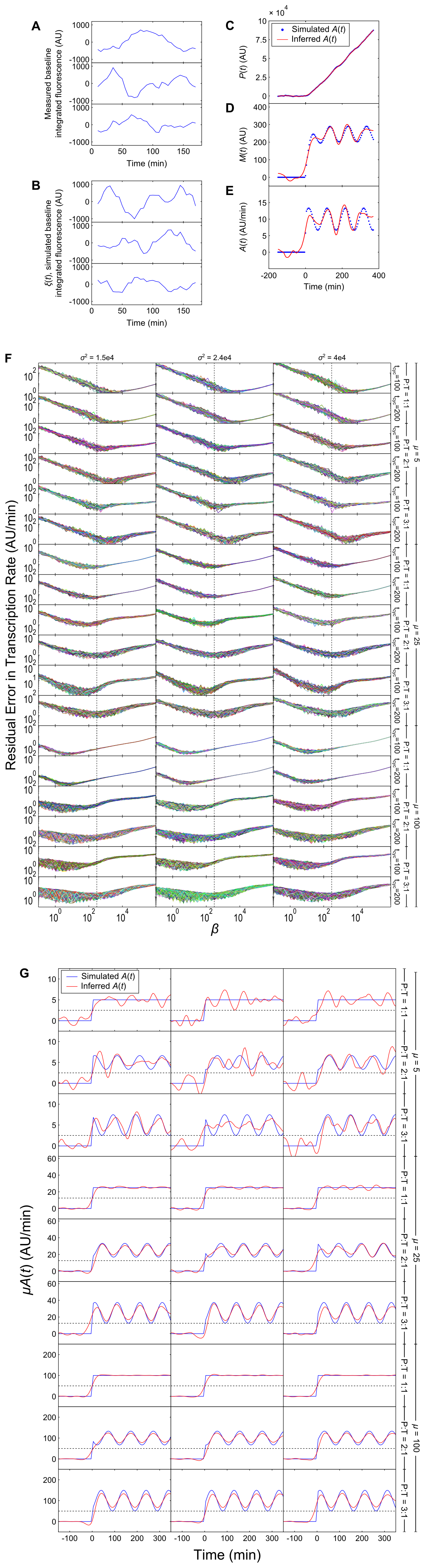

Supplement: Figure S15 — Simulation of realistic total protein time series to determine optimal smoothing parameter for spline fit. Using the method described in Text S1, noise characteristics of experimental integrated fluorescence data before splining (A, representative single cell traces from the P1xtetO and P7xtetO step tests) were used to simulate baseline protein time series (B). (C–E) A(t) was simulated for a step up at t = 0 from zero to an oscillating steady state with the period of a typical cell cycle (E, tcyc = 100 min, P∶T = 2∶1). A simulated mRNA (D, μ = 10) and total protein trace (C) were calculated from A(t) using Eqns. (S5–8), with noise added to the protein time series ξ(t) similar to the high-frequency, measurement noise observed in data (σ 2 = 2.4e4). A smoothing spline was fit to the simulated protein time series and the corresponding mRNA and transcription rate time series were inferred using Eqns. (1–2) in the Methods. (F) The simulation was repeated for all combinations of parameters described in the text that span observed characteristics of protein time series across all experiments. For each parameter set (panels), 100 noisy, asynchronous traces were generated and the residual error between the simulated and the inferred transcription was found across a range of smoothing parameters β (colored lines). The chosen β = 300 (vertical, dashed line) minimizes the residual error across all parameter sets and was used for all data sets. (G) Sample comparisons of simulated and inferred transcription rate μA(t) (using β = 300) for each parameter set with tcyc = 100 min. Black dotted line represents the half the steady state average for t>0 (the threshold for determining activation in a step test). (TIF) [file pcbi.1003161.s015.tif]

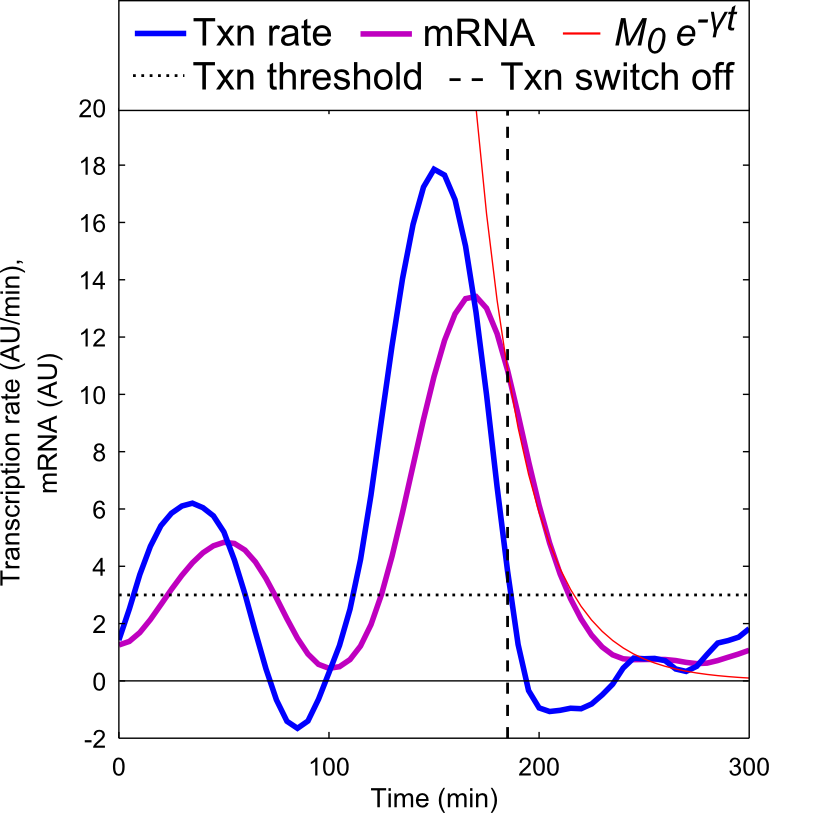

Supplement: Figure S16 — mRNA levels fall exponentially with the expected degradation rate after periods of strong transcription. For the single cell in the bottom panel of Figure S6, the CFP transcription rate (blue) and mRNA level (purple) show sporadic transcription at low expression levels. At the end of a strong “on” period (vertical, dashed line), where transcription rate drops below the threshold (horizontal, dotted line), the inferred mRNA level drop is consistent with the expected first-order degradation process (red line), where γ is the mRNA degradation rate and M0 is a constant. (TIF) [file pcbi.1003161.s016.tif]

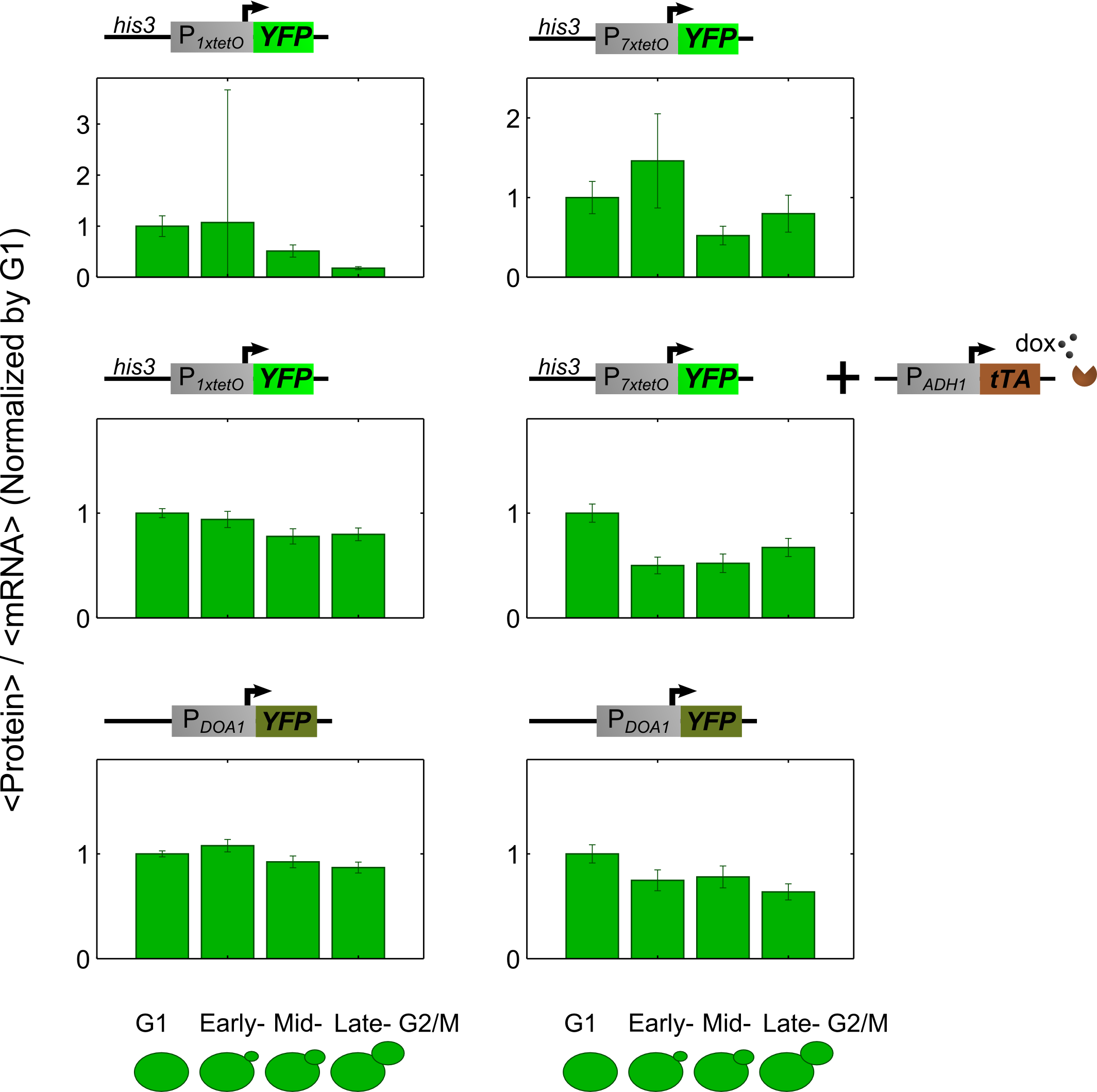

Supplement: Figure S17 — Translational capacity is not cell-cycle dependent. The average protein to mRNA ratio of cells grouped by cell-cycle phase (based on bud size) is shown for P1/7xtetO and PDOA1. This ratio exhibits no clear cell-cycle dependent trend, indicating that cell-cycle dependent changes in translation rate are not a dominant source of changes in protein level across the cell-cycle. Error bars are s.d. obtained by bootstrapping. (TIF) [file pcbi.1003161.s017.tif]

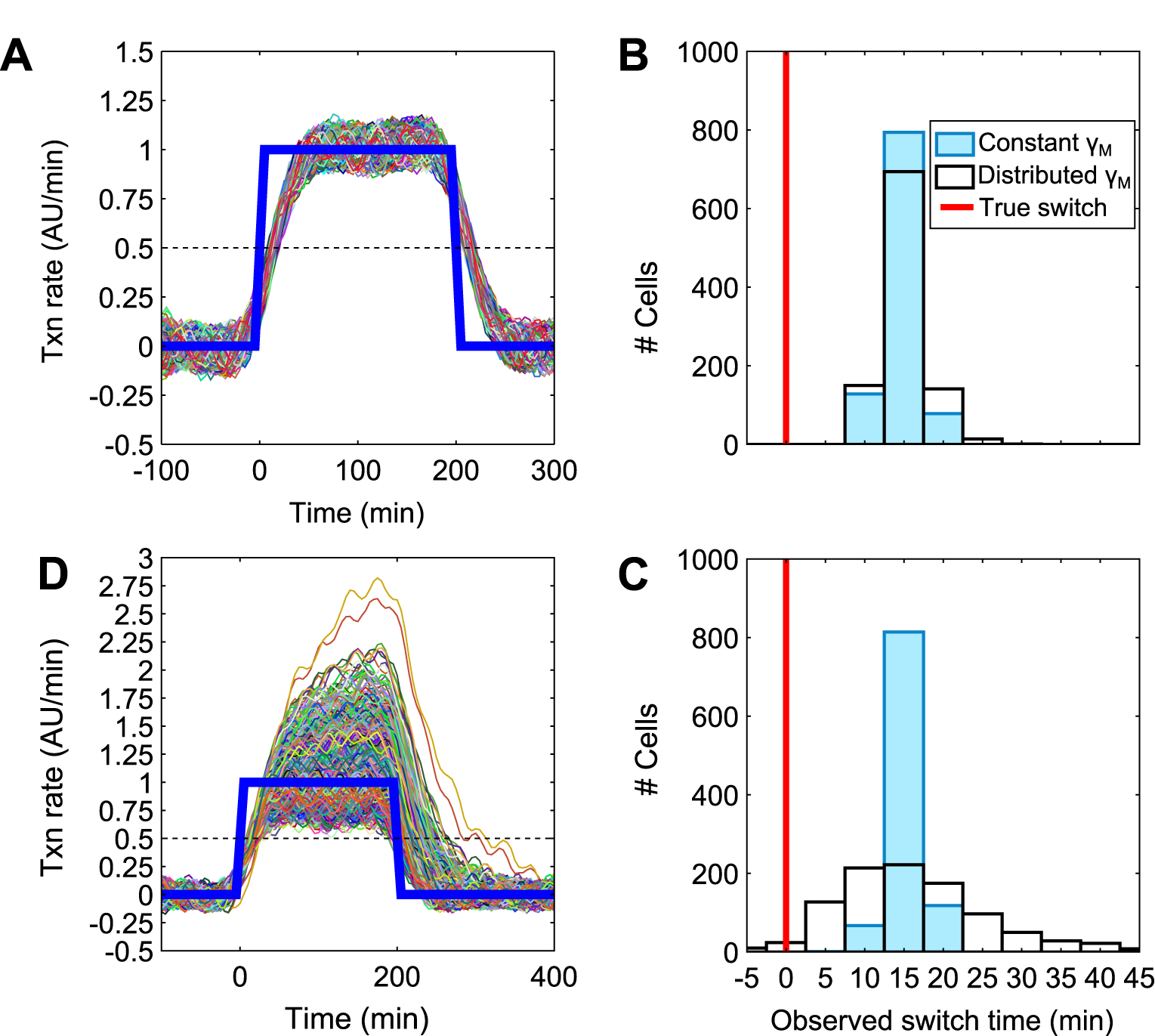

Supplement: Figure S18 — Simulations to estimate the time accuracy of the transcription rate transition time assignments. (A) Transcription rate time series (thin colored lines) are inferred for noisy protein data simulated from the square pulse transcription rate generator function (thick, blue line) as in Figure S15 (n = 1000). Here a protein maturation step is included in simulating the protein data, but not in the transcription rate inference. (B) Activation and (D) deactivation times “observed” for each inferred transcription trace in A are delayed 10–15 min relative to the true times (blue histogram compared to vertical, red line) with a mean absolute deviation of ∼2 min. (C) Allowing the mRNA degradation rate γM in Eqn. (S6) to vary during simulation, but inferring transcription rate using the single, average γM value, results in greater dispersion in the sample traces. The “observed” activation times did not vary much more, but deactivation time mean absolute deviation increased to ∼8.4 min (black histograms in B and D). (TIF) [file pcbi.1003161.s018.tif]
