# Supplementary material for: Cell-Cycle Dependence of Transcription Dominates Noise in Gene Expression
Source: PLoS Comput Biol. 2013 Jul 25;9(7):e1003161. doi: 10.1371/journal.pcbi.1003161 (PMC3723585; doi:10.1371/journal.pcbi.1003161)
Supplement: Table S1 — Yeast strains used in this study. (PDF) [file pcbi.1003161.s019.pdf]

Table S1. Yeast strains used in this study

Strain	Relevant Genotype	Parent Strain	Reference
Y1	*MAT**a** trp1-1 can1-100 leu2-3,112 his 3-11,5 ura3 GAL+ ADE+*	W303	Laboratory collection
Y3	*MAT**a** ade2-1 trp1-1 can1-100 leu2-3,112 his 3-11,5 ura3 GAL+*	W303	Laboratory collection
Y6	*MAT**α** ade2-1 trp1-1 can1-100 leu2-3,112 his 3-11,5 ura3 GAL+*	W303	Laboratory collection
Y47	*MAT**a** leu2::*P_{ADH1}*-CFP-hisG::URA3::kanR::hisG*	Y1	Gift from E. O'Shea [26]
Y139	*MAT**a** his3::*P_{1xtetO}*-vYFP-HIS3*	Y1 +B163	Laboratory collection
Y163	*MAT**a** his3::*P_{7xtetO}*-vYFP-HIS3*	Y1 +B165	Laboratory collection
Y216	*MAT**α** ade2-1 ura3::*P_{7xtetO}*-vYFP-kanR*	Y6	Laboratory collection [27]
Y231	*MAT**a** leu2::*P_{PGK1}*-RFP ADE2::*P_{MYO2}*-tTA ura3::*P_{7xtetO}*-CFP-kanR*	Y3	Laboratory collection [27]
Y236	*MAT**a/α** leu2::*P_{PGK1}*-RFP ADE2::*P_{MYO2}*-tTA ura3/ura3::*P_{7xtetO}*-CFP-kanR/* P_{7xtetO}*-vYFP-kanR*	Y231xY216	This study
EY2210	*MAT**a** ADE+ NHP2::RFP-NAT spl2Δ::LEU2 pho4Δ::PHO4(1-741bp)-cYFP-HIS3 pho84Δ::CFP-kanR*	W303	Gift from E. O'Shea
EY2150	*MAT**a** ADE+ NHP2::RFP-NAT spl2Δ::LEU2 pho84Δ::CFP-kanR*	W303	Gift from E. O'Shea

	PHO4::cYFP-HIS3		
EY131	*MAT**a** pho4Δ::TRP1*	W303	Gift from E. O'Shea
Y114	*MAT**a** ADE+ NHP2::RFP-NAT spl2Δ::LEU2 pho84Δ::CFP-kanR pho4Δ::TRP1*	EY2210 (EY131 PCR template)	This study
Y152	*MAT**a** ADE+ NHP2::RFP-NAT spl2Δ::LEU2 pho4Δ::TRP1 pho84Δ::CFP-kanR phm4Δ::*P_{GPD}-*PHO81-HIS3MX6 leu2Δ::*P_{TEF1m7}-*PHO4-cYFP*	Y114 (+ B133 PCR product, + B141)	Laboratory collection
Y320	*MAT**a** ADE+ NHP2::RFP-NAT spl2Δ::LEU2 pho4Δ::TRP1 pho84Δ::KlURA3*	Y114	This study
Y532	*MAT**a** doa1::vYFP* + pRS316-*DOA1*	Y1 + B858	This study
Y784	*MAT**a** ADE+ NHP2::RFP-NAT spl2Δ::LEU2 pho4Δ::TRP1 pho84Δ::klura3*	Y320	This study
Y785	*MAT**a** ADE+ NHP2::RFP-NAT spl2Δ::LEU2 pho4Δ::TRP1 pho84Δ::klura3 phm4Δ::HIS3MX6*	Y784	This study ("kinetic" base strain)
Y947	*MAT**a** ADE+ NHP2::RFP-NAT spl2Δ::LEU2 pho4Δ::TRP1 pho84Δ::klura3 phm4Δ::HIS3MX6 leu2Δ::*P_{TEF1m7}-*PHO4ΔP2-tetR-cYFP*	Y785 (+B798)	This study

Y955	*MAT**α** his3::*P$_{1xtetO}$*-tdTomato-HIS3*	Y2 (+B229)	This study
Y960	*MAT**a** ADE+ NHP2::RFP-NAT spl2Δ::LEU2 pho4Δ::TRP1 pho84Δ::klura3 phm4Δ::HIS3MX6 leu2Δ::*P$_{TEF1m7}$*-PHO4ΔP2-tetR-cYFP URA3::*P$_{1xtetO}$*-CFP*	Y947 (+B579)	This study
Y962	*MAT**a** ADE+ NHP2::RFP-NAT spl2Δ::LEU2 pho4::TRP1 pho84Δ::klura3 phm4::HIS3MX6 leu2Δ::*P$_{TEF1m7}$*-PHO4ΔP2-tetR-cYFP URA3::*P$_{7xtetO}$*-CFP*	Y947 (+B720)	This study
Y1011	*MAT**a/α** his3/his3::*P$_{1xtetO}$*-vYFP-HIS3/*P$_{1xtetO}$*-tdTomato-HIS3*	Y139 x Y955	This study
