# Supplementary material for: Cell-Cycle Dependence of Transcription Dominates Noise in Gene Expression
Source: PLoS Comput Biol. 2013 Jul 25;9(7):e1003161. doi: 10.1371/journal.pcbi.1003161 (PMC3723585; doi:10.1371/journal.pcbi.1003161)
Supplement: Table S2 — Plasmids used in strain construction. (PDF) [file pcbi.1003161.s020.pdf]

Table S2. Plasmids used in strain construction

Plasmid	Base vector	Relevant gene	Construction information
B48	Yeast centromeric	P_{1xtetO}-*CFP*	Laboratory stock
B89	pJRL2	P_{PGK1}-*vYFP*	Laboratory stock, *leu2* integrating vector [26]
B108	pBluescript	*KlURA3*	Laboratory stock, contains *URA3* gene from *K. lactis*; PCR template for *pho84Δ* in Y320
B129	pRS303	*HIS3MX6*	*HIS3MX6* from pFA6a-*HIS3MX6* [28] (BglII/EcoRI digested) ligated into pRS303 (BamHI/EcoRI); PCR template for *phm4*Δ in Y785
B133	pRS303	P_{GPD}-*PHO84*-*HIS3MX6*	Into pRS303, cloned P_{GPD}-*PHO84* (SalI/PvuII into SalI/EcoRV) and *HIS3MX6* (BglII/EcoRI into BamHI/EcoRI)
B141	pJRL2	P_{TEF1m7}-*PHO4* -*cYFP*	PCRed *PHO4-Citrine* from EY2150 genome and clone P_{TEF1m7} (SalI/EcoRI) – *PHO4 – Citrine* (EcoRI/NotI) into SalI/NotI digested pJRL2 (B89). P_{TEF1m7} from [29].
B163	pRS303	P_{1xtetO}-*vYFP*	Laboratory stock
B165	pRS303	P_{7xtetO}-*vYFP*	Laboratory stock
B198	pRS303	P_{7xtetO}-*tTA*	Laboratory stock
B228	pCM189	P_{ADH1}-*tTA*	Laboratory stock

B229	pRS303	P_{1xtetO}-*tdTomato*	Laboratory stock
B464	pBluescript	*klura3*	Digested B108 with EcoRV and self-ligated to remove portion of ORF and make *KlURA3* non-functional; PCR template for *klura3* Y784
B579	pRS306	P_{1xtetO}-*CFP*	XhoI/NotI digested P_{1xtetO}-*CFP* from B48 ligated in front of T_{ACT1} in pRS306 (XhoI/NotI)
B720	pRS306	P_{7xtetO}-*CFP*	XhoI/BamHI digested P_{7xtetO}-*CFP* from B198 ligated in front of T_{ACT1} in pRS306 (XhoI/NotI)
B798	pJRL2	P_{TEF1m7}-*PHO4ΔP2-tetR-cYFP*	P_{TEF1m7} (SalI/EcoRI) – *PHO4*(1-600bp) (EcoRI/SpeI) – linker – *tetR* (SpeI/PacI) – linker – *Citrine* (PacI/NotI) ligated into SalI/NotI digested pJRL2 (B89)
B858	pRS316	P_{DOA1}-*DOA1*	PCRed *DOA1* locus from Y1 (BamHI/NotI) and ligated into pRS316
