# Supplementary material for: Cell-Cycle Dependence of Transcription Dominates Noise in Gene Expression
Source: PLoS Comput Biol. 2013 Jul 25;9(7):e1003161. doi: 10.1371/journal.pcbi.1003161 (PMC3723585; doi:10.1371/journal.pcbi.1003161)
Supplement: Table S3 — Comparing experimental mRNA expression distributions to simple models. (PDF) [file pcbi.1003161.s021.pdf]

Table S3. Comparing experimental mRNA expression distributions to simple models

	A	B	C	D	E	F
	**tetO promoter without activator (basal)**		**tetO promoter with tTA activator**		**Constitutive DOA1 promoter**	
	**1xtetO****	**7xtetO*****	**1xtetO**	**7xtetO**	**Higher**	**Lower**
**Ratio of mean mRNA at late G2/M to G1 as a measure of fold-change in transcription:**						
$\hat{R}_M$ (measured)	9.3	3.1	2.8	2.7	2.4	4.2
Theoretical corresponding *f*	Inf	4.3	3.6	3.3	2.7	8.9
**Negative binomial fit to total distribution:** The standard model equates a frequency and size of bursting to parameters of negative binomial fit to total population's stationary distribution.						
**"Burst frequency"**	**0.4**	**0.5**	**2.2**	**1.2**	**4.1**	**1.1**
**"Burst size"**	**7.7**	**8.5**	**5.8**	**10.0**	**1.4**	**1.3**
**2-fold transcription increase: *** Poisson transcription with S/G2/M transcription rate increased 2-fold over G1 transcription rate. The transcription rate is fit to the experimental total mean mRNA count.						
f	**2**	**2**	**2**	**2**	**2**	**2**
χ^2 fit p-value, Total	0	0	0	0	0.95	0.96
χ^2 fit p-value, G1	0	0	0	0	0.02	0.002
χ^2 fit p-value, G2	0	0	0	0.001	0.001	0.001

***f*-fold transcription increase: ***<br>Poisson transcription with S/G2/M transcription rate increased *f*-fold over G1 transcription rate.<br>The transcription rate and *f* are fit to the experimental G1 and G2 mean mRNA count.						
f	**Inf**	**100**	**4**	**9**	**4**	**12**
χ^2 fit p-value, Total	0.06	0.002	0.14	0.07	0.004	0.12
χ^2 fit p-value, G1	0.69	0.17	0.46	0.84	0	0.002
χ^2 fit p-value, G2	0.004	0	0.18	0.007	0.78	0.69
***f*-fold transcription increase with variable timing of transition: ***<br>Poisson transcription with S/G2/M transcription rate increased *f*-fold over G1 transcription rate with a 40 minute uniformly distributed window of switching to S/G2/M transcription rates after the S/G1 transition.<br>The transcription rate and *f* are fit to the experimental G1 and G2 mean mRNA count.						
F	**Inf**	**100**	**6**	**13**	**7**	**18**
χ^2 fit p-value, Total	0.85	0.003	0.13	0.15	0	0
χ^2 fit p-value, G1	0.35	0.06	0.30	0.73	0	0
χ^2 fit p-value, G2 *	0.01	0	0.84	0.76	0.06	0.27

* Result of a χ^2 goodness of fit test of the measured data against the model prediction. Cases that pass or fail are in black or gray text, respectively with p = 0.05 representing the cutoff.

** For basal expression from P_{1xtetO}, the G1 and S/G2/M expression differ more than expected for no transcription in G1. Thus the data is best fit where *f* = infinity, representing no G1 transcription.

*** For basal expression from P_{7xtetO}, because of high expression noise and the fact that 10% of cells have not turned on by late G2/M, specifying *f* by matching G1 and S/G2/M means gives a value of *f* (~7) that fits distributions poorly. The χ^2 goodness of fit was maximal and fairly constant over a range of approximately $50 < f < 100$. Thus, *f* = 100 was selected. For the model with variable timing of transitioning to G2 transcription rates, the estimated 40 minute window was extended to the full duration of S/G2/M.
