# Supplementary material for: Cell-Cycle Dependence of Transcription Dominates Noise in Gene Expression
Source: PLoS Comput Biol. 2013 Jul 25;9(7):e1003161. doi: 10.1371/journal.pcbi.1003161 (PMC3723585; doi:10.1371/journal.pcbi.1003161)
Supplement: Text S1 — Supplementary methods and discussion. (DOCX) [file pcbi.1003161.s022.docx]

Text S1

Contents

Supplementary Methods 2

The “kinetic” strain to measure activation delay 2

Fluorescent protein maturation measurement 3

Time series analysis 5

Extracting single-cell volume and fluorescence time series from microscopy movies 5

Spline-fitting to differentiate time series 7

Determining thresholds for activation of transcription 11

Time accuracy of the calculated transcription transition times 12

Supplementary Discussion 14

Translational capacity and cell-cycle 14

Stochastic models of cell-cycle dependent transcription for P_1/7xtetO_ and P*_DOA1_* 15

Intrinsic noise in tetO promoter expression 17

Supplementary References 18

# **Supplementary Methods**

## The “kinetic” strain to measure activation delay

We reengineered the phosphate starvation pathway to meet three requirements: 1) the master transcription factor (TF) Pho4p had to rapidly localize into and out of the nucleus in response to changing extracellular phosphate concentrations, 2) the high and low phosphate concentrations needed to toggle Pho4p localization would remain high enough so changing intracellular phosphate concentration had no effect on growth, and 3) Pho4p variants could be generated that target specific promoters. Normally, cells employ a dual positive and negative feedback loop architecture to respond to changes in external phosphate concentration. This results in switching the complement of high and low affinity phosphate transporters present on the membrane and can even result in a bimodal population response at intermediate levels of phosphate [1]. Our strategy was to eliminate these feedback loops and retune the system to meet our criteria. We started with strain EY2210 [1]. The *spl2Δ* prevents down regulation of low affinity phosphate transporters in low phosphate conditions and hence eliminates the positive feedback loop. Also *PHO84*, encoding the high affinity transporter, is replaced with CFP. With no high affinity phosphate transporter, which normally is upregulated in low phosphate conditions [2], there is no negative feedback loop. This strain also expresses an Nhp2p-mCherry protein fusion. Because we sought to look at CFP expression from tetO promoters, we replaced CFP at the *PHO84* locus with a *KlURA3* (using a PCR fragment from B108) and regenerated uracil auxotrophy by eliminating a portion of *KlURA3* (using a short PCR fragment from B464). Because utilization of vacuolar stores of phosphate can delay the response to changes in external levels [3], we eliminated a key vacuolar transporter, Phm4p, by integrating a *phm4Δ::HIS3MX6* PCR fragment. In this background, growth in the microfluidic chamber in low external phosphate (5 μM) slows relative to growth in high phosphate (5000 μM) (Figure S10); however, this occurs gradually and does not significantly impact either changes in Pho4p’s nuclear localization or the transcription rate on the timescale of the experiments (Fig. 5). (While 5 μM external phosphate leads to a severe growth defect in culture and is reportedly below the *K_m_* of the low affinity transporters [4], we have found both nutrients and drugs are more potent in the microfluidic chamber. This is likely due to both constant perfusion and a reduction in mass transfer limitations present in culture). Finally, we deleted the endogenous *PHO4* using a *pho4Δ::TRP1* PCR fragment (from EY131 genome, kind gift of E. O’Shea), and introduced Pho4p-tetR-YFP at the *leu2* locus. The C-terminal Pho4p DNA binding domain is well-defined [5] and easily eliminated [6], so we replaced it with a tetR C-terminally fused to Citrine, a YFP variant [7]. YFP was fused to tetR using the peptide linker (GDGAGLIN) reported in [8], but modified for more common codon usage and to convert the PacI restriction site to a SpeI site by exchanging valine for isoleucine (GGT GA**T** GGT GCT **GGA** **CTA** **GTT** AA**T**, translated as GDGAGL**V**N). The modified linker was then used to fuse tetR to the *PHO4* shuttling domain (bp 1-600). Because Pho4p is known to dimerize with the Pho2p cofactor we also deleted the Pho2p binding domain in *PHO5* (bp 601-741) [9], further insulating the pathway from native phosphate regulation. Into the resulting strain (Y947), we integrated either P*_1xtetO_* or P*_7xtetO_* driving CFP at the *URA3* locus using integrating plasmids (B579 or B720) (Fig. 5A). Genotypes were verified at each stage in construction by PCR.

## Fluorescent protein maturation measurement

We measured the maturation rate of CFP (Cerulean), YFP (Venus), and RFP (tdTomato) using the 3-color strain similar to the method of [10]. Cells were cultured to mid log-phase growth, loaded into the microfluidics, and allowed to grow for 3 hrs. Then the flow was switched to media containing 30 μg/mL cycloheximide to block translation and images were acquired every 5 minutes. Initially the fluorescence increased, presumably due to maturation of the remaining immature protein pool. CFP and YFP fluorescence peaked quickly before a slow decline (Figure S12A). The loss of observable fluorophore occurs at longer time-scales and is most likely due to fluorophore bleaching because the fluorescent proteins are very stable. We fit the measured data to a simple model describing both maturation and loss of observable protein, *P*:

Here, *I* is the immature protein, *k_m_* is the maturation rate and *γ_P_* is the loss rate, probably mostly photobleaching. The time-dependence of the observable protein can be easily determined after cycloheximide addition:

$$P(t)=\frac{k_{m}I_{0}}{\gamma_{P}-k_{m}}\left(e^{-k_{m}t}-e^{-\gamma_{P}t}\right)+P_{0}e^{-\gamma_{P}t}$$

Here, *I_0_* and *P_0_* are the amounts of immature and mature protein at the time of cycloheximide addition. We fit Eqn. (S3) to the CFP and YFP time series for each cell starting at the time of cycloheximide addition to obtain values for *I_0_*, *P_0_*, *k_m_*, and *γ_P_* for each trace. For RFP, the time-series never decreased, suggesting over the time of the experiment the maturation process was dominant over photobleaching (Figure S12A). Therefore, we fit the RFP time series assuming *γ_P_* = 0 (reducing the fit to a single exponential). The median maturation half-lives (ln(2)/*k_m_*) for CFP, YFP, and RFP were found to be 10, 32, and 150 min, respectively (Figure S12B). The corresponding loss/bleaching half-lives (ln(2)/*γ_P_*) for CFP and YFP were 38 and 10 hrs, respectively. If some bleaching of RFP occurs, the 150 min maturation half-life represents a lower bound.

Unfortunately, we are not certain about the accuracy of these estimates for several reasons. First, on average CFP and YFP traces appear to change identically in response to cell-cycle dependent changes in transcription, within a multiplicative constant. This is true not only for situations of high expression with transcription occurring in both G1 and S/G2/M, but also with low expression where active transcription is only observed in S/G2/M. Given the difference in maturation, one would expect a larger delay in YFP expression. Second, we have performed step tests like those in Fig. 5 in a diploid variant of the kinetic strain expressing both CFP and YFP from a responsive promoter. The CFP and YFP responses are again identical within a multiplicative constant. Third, the fit value of the ratio of immature to mature protein initially ($I_{0}/P_{0}$) is 0.10 and 0.33 for CFP and YFP, respectively. While this is qualitatively consistent with YFP having a slower maturation rate, it is quantitatively unreasonable. Cells are expressing CFP and YFP at steady-state levels before cycloheximide addition, so $I_{0}/P_{0}=\gamma_{P}/k_{m}$. Using the inferred maturation and loss/bleaching rate yields an expected $I_{0}/P_{0}$of 0.005 and 0.05 for CFP and YFP. Fourth, the long maturation time of RFP should in principle dramatically smooth cell-cycle dependent transitions in the RFP trace, and yet we and others [11] observe these transitions.

Because of the uncertainties in maturation, we chose to analyze the time series data using Eqns. (1&2) (Methods) which do not account for maturation. We discuss in detail how including maturation affects our results below. Because we have observed both YFP and CFP expression within 15 minutes of turning a gene on (using the kinetic strain), we suggest the maturation rate is similar and closer to the value measured for CFP (10-15 minute half-life). Importantly, the central findings in the main text that (i) transcription rate is greater in S/G2/M versus G1, (ii) at low expression levels transcription can be restricted to S/G2/M, and (iii) transcription activation delays are shorter in S/G2/M relative to G1 are qualitative and robust to changes in the maturation rate.

## Time series analysis

### Extracting single-cell volume and fluorescence time series from microscopy movies

Microscopy movies for each acquisition channel (plus a nuclear mask movie from thresholding the RFP movie when the kinetic strain was used) were compiled in MetaMorph for analysis in Matlab (MathWorks, Natick, MA). We wrote a custom GUI-driven package for semi-automated image processing that segmented cells, defined budding times, and tracked cell lineages, yielding time series for volume and protein concentration (as measured by average fluorescence) (Fig. 1C-&D, main text).

Pre-processing movies consisted of registering and segmenting cells. To account for imprecise return to each stage position at successive time points, movies were first registered using a 2D cross-correlation of BF images between time *t* and *t*+1. Segmentation relied on the large differences in contrast of the cell wall between a BF (dark cell with light cell wall ring) and BFOOF (light cell with dark outer ring) images, and involved a series of morphological operations and watershedding to identify single cell regions. A tracking and visualization GUI used a Matlab implementation of an IDL Particle tracking routine (Blair and Dufresne, available at: <http://physics.georgetown.edu/matlab/>) to assign cell IDs in each frame based on following region centroids. When a bud appears (new region and ID), the corresponding mother is assigned from the nearest neighbor regions. Lineage assignments for each bud are optimized by penalizing potential mothers based on distance between bud and mother perimeters, and based on potential matches to other buds in nearby times. The GUI then allows rapid visual inspection and manual curation of the accuracy (90-95% of the data) of automated region segmentation, tracking IDs, and lineage assignments before finalizing cell measurement time series.

For each single cell region, we estimated raw cell volume similar to [12]: as an ellipsoid of cross-section corresponding to the region and constrained by the microfluidic trapping chamber, but with mothers and buds treated separately. We interpreted the average pixel intensity in each region as a raw volumetric concentration (Fig. 1D) because the depth of field (0.5 μm) was significantly less than the trapping chamber height (3.5 μm) and the fluorescence profile across the cell was flat, rather than the elliptical profile expected if light were captured from the entire cell volume. Both volume and concentration time series for each cell were conservatively smoothed to remove measurement noise using the spline method described below; total protein was estimated as the product of the two (Fig. 1D). Though volume and total protein estimation of buds occurred separately from mothers, the data from the bud-mother pair were summed until cytokinesis to represent the whole cell (Fig. 1C&E). Although we do not account for uncertainties in cytoplasmic volume due to volume of organelles such as vacuoles and the nucleus, the rise in total fluorescence clearly accelerates after budding even after averaging across many cells (Fig. 2B, main text) so the calculated acceleration in protein production is not an artifact. The online protocol contains further details of the automated algorithms used.

We used morphological cues to define transitions from G1 to S/G2/M. Yeast cells pass through START to transition to S phase, which occurs slightly before a bud appears [13], so we estimated the time of bud appearance to approximate the G1/S transition. To do so, we linearly extrapolated the bud volume trace back to zero volume using the bud volume measured at the first five time points the bud was successfully segmented. Clear morphological markers for cytokinesis (the M/G1 transition) were the bud neck narrowing after nuclear division (easily observable when the nucleus is fluorescently labeled) and the formation of a dark line between the mother and daughter (bud) in a bright field image (Figure S1). These events strongly correlated with the end of a brief plateau in the bud volume, providing an easy way for automated identification based on the slope in the bud volume trace (Fig. 1C).

### Spline-fitting to differentiate time series

We sought to estimate mRNA number and identify transitions from an inactive to active promoter state by examining rates of change in the protein time series. While such transitions might be more apparent from direct measurements of an unstable protein, its rapid degradation makes detection difficult at low levels of expression. Instead, we developed a method to estimate first and second time derivatives of the measured volume and fluorescence time series and thereby infer the transcription rate. Because of noise in experimental data, estimating time derivatives is an ill-posed problem, and direct methods (e.g., finite differences) will amplify the noise with each application. Local splining techniques such as the Savitzky-Golay method [14] can yield a smooth first derivative for noisy data, but will amplify noise for higher order derivatives required in our subsequent analysis. We instead fit each time series to a cubic smoothing spline following the method of de Boor [15]. This algorithm calculates a single continuous spline, balancing accuracy and smoothness by minimizing a linear combination of the least square errors of the fit and the spline’s second derivative (a measure of roughness). A single smoothing parameter, *ρ*, determines the relative weight in the minimization between the residuals and the curve roughness to allow an appropriate smoothing of noise in the data, *y*, while preserving real features. The resulting smoothing spline, *f*, then minimizes the expression

$$\rho\sum_{j=1}^{n} w(j)\left|y(j)-f(x(j))\right|^{2}+(1-\rho)\int\left|D^{2}f(t)\right|^{2}dt$$

where *x* are the corresponding times for the data points *y*, with *w* the relative error weighting for each point.

We applied the MATLAB “csaps” implementation of this method to time series for each cell in two stages each with its own smoothing parameter. Due to the sensitivity of the spline fit to *ρ*, a suitable *ρ* is often near $1/(1+\Delta t^{3}/6*\beta)$ (as noted in the Matlab documentation for the “csaps” function), and so we approach spline smoothing by setting a suitable β. In the first stage, we fit a spline to both the volume and concentration time series to correct outlying data caused by large autofocusing and segmentation errors. Using a heuristically-chosen, conservative smoothing parameter (β = 3), the raw data was pre-treated to facilitate budding and division time assignments as well as the construction of integrated fluorescence time series, computed as the product of the volume and concentration splines (Fig. 1E, main text).

In the second stage, the total fluorescence and total volume time series (the sum of mother and bud series) were again fit to a spline using a second smoothing parameter chosen to provide reliable time-derivatives for each. An ideal β would result in a spline *f* that minimizes fluctuations in the derivatives due to noise in the measurement but preserves real features in the derivative time series. This β depends on the characteristics of both noise and real features in a particular data series, but it is difficult to distinguish these *a priori* for any one condition. We therefore chose to fit all fluorescence time series to a single, pre-determined β. By treating all data consistently, we maintain clarity in interpretation across all experiments with the understanding that series with fewer and smaller real features will yield slightly noisier splines. A computational strategy allowed the determination of a single, suitable β to spline total fluorescence for the different experimental conditions (also suitable for total volume, which was much less sensitive to the choice of smoothing parameter).

First, we postulate typical features in a transcription rate time series, and then computationally generate representative mRNA and protein time series from that transcription rate. The prominent features we include in the simulated transcription rate are informed by experimental observations: cell cycle-dependent oscillations, different expression levels, and sharp transitions. Total protein accumulation accelerates after budding (main text, Fig. 2B), so we simulate the transcription rate in an oscillatory manner with a period of the cell cycle and an arbitrary mean of 1:

$$A_{sim}(t)=u(t)\left[\alpha\sin\left(2\pi\left(\frac{t}{t_{cyc}}+\phi\right)\right)+1\right]$$

where *α* is a scaling factor to set the peak to trough ratio (P:T) and *t_cyc_* is the doubling time. We vary P:T from 1:1 to 3:1 when setting *α* to simulate behavior from flat transcription to a cycle-dependence greater than gene dosage. The cycle length *t_cyc_* is either 100 or 200 min, the approximate bounds for growth in glucose or raffinose. The inclusion of *u*(*t*), the Heaviside step function, reflects the sharp transitions present in step tests. This is included in all simulations to combine transient and steady-state transcription behavior in each fit. Finally, to generate multiple asynchronous time series akin to the cell population we measure, we include *φ* as a random number from 0 to 1 to randomize the initial cell cycle position. Solving Eqn. (2) in the Methods for *M*(*t*) yields:

$$M_{sim}(t)=\mu\left(u(t)e^{-\gamma_{M}t}\int e^{\gamma_{M}t}A_{sim}(t)dt\right)$$

with *μ* a transcription rate scaling factor that depends on promoter strength, and varies from 1 to 100 to match observed expression levels. *γ_M_* is the mRNA degradation rate, set to 0.04 min^-1^ measured in [16]. The total protein can likewise be computed by solving Eqn. (2) in the Methods for *P*(*t*):

$$P_{sim}(t)=k_{t}\int M_{sim}(t)dt+\xi(t)$$

where *k_t_* is the translation rate (a redundant scaling factor kept at 1 here), and *ξ*(*t*) adds high-frequency measurement noise in total protein so that we may determine the effect of measurement errors on choosing β.

The noise term *ξ*(*t*) models the noise observed in the total fluorescence measurement after the first splining stage when there is no transcription – i.e., a baseline level (Figure S15A). Baseline fluorescence time series could be experimentally obtained using the kinetic strain under high phosphate conditions when the transcriptional activator is cytoplasmic, transcription does not occur, and the fluorescence level corresponds to cellular autofluorescence. We describe this baseline noise– the product of smoothing raw, high-frequency noise in the volume and concentration time series – using an autoregressive-moving average (ARMA) model. This parametric approach allows us to generate stochastic baseline noise reflecting the statistics of experimental observations. To each of 86 representative baseline traces, we fit a 4^th^ order ARMA model:
$\xi(t)+a_{1}\xi(t-1)+...+a_{4}\xi(t-4)=e(t)+c_{1}e(t-1)+...+c_{4}e(t-4)$
where the coefficients *a_i_* and *c_i_* define the autoregressive and moving average polynomials, respectively, and *e*(*t*) is Gaussian white noise time series with variance *σ*^2^. To define our noise term *ξ*(*t*) as a representative ARMA(4,4) model, we take the median of all fits for each coefficient. This model then allows us to generate unique baseline noise traces with representative statistics simply by simulating a unique *e*(*t*) (Figure S15B). To ensure we capture a realistic range of noise strength in the simulations, we vary *σ*^2^ by setting it to either the 25^th^, 50^th^, or 75^th^ percentile of the fit values.

Next, we fit a spline to each simulated protein time series over a range of values for β to infer the underlying transcription rate (using Eqns. (1-2), Methods; Figure S15C-E). We compared this inferred rate for each β to the initially postulated *A_sim_*(*t*) and determined the β corresponding to the minimum residual error. To be sure our chosen β is suitable for splining all the data, the parameters *α*, *t_cyc_*, *μ*, and *σ*^2^ are varied within the ranges given above to generate noisy protein time series reflecting the gamut of experimental data. For each combination of the parameters shown in Figure S15F, 100 protein time series with a random instance of *φ* and *ξ*(*t*) were generated, and the inferred, underlying transcription rate error was calculated for the range of β shown. The residual-minimizing β is insensitive to the parameters *t_cyc_*, and *α* over the ranges encountered in the experiments; however, the magnitude of the noise term (*σ^2^*) relative to the expression level (*μ*) strongly influences the range of β that provides an accurate inference of transcription rate. Predictably, relatively noisier protein traces require a higher β for satisfactory smoothing, while stronger transcription (i.e.*,* stronger features) can be more accurately inferred at lower β. Therefore, there is no single β that will perfectly infer transcription at the noisiest baseline levels (upper right panels) and at the clear, strongest levels (lower left panels), but these simulations inform our decision in making this inherent tradeoff.

The important biological observation of cell cycle-dependent transcription relies not on quantitative accuracy in the inferred transcription rate but on correct inference of the *timing of* *transitions* in transcription rate. Undersmoothing preserves the sharpness of transitions, but noise is amplified in derivatives, possibly indicating false starts and stops in transcription. On the other hand, oversmoothing preserves transition times on average, but individual transitions are degraded in a trace-dependent manner. For this reason, a β of 300 was heuristcically chosen to most accurately infer transcription rate for the range of scenarios in Figure S15F. This β best preserves transition times at the expense of tracking some noisy fluctuations during periods of gradual change and eroding the edges of the sharpest features (Figure S15G).

Two additional properties of experimental time series data must be taken into account when using spline fits to estimate derivatives. First, there is limited data at the beginning and end of a time series. To address this, we tapered the first and last three weights in *w* to diminish edge effects on nearby derivatives (all interior points were weighted equally). Second, sharp jumps in data across cell divisions lead to undefined second order derivatives. Therefore, the time series across a mother/daughter division was extended by maintaining a continuous whole cell “running total” of volume and fluorescence of the mother even after division (Fig. 1C&E). This allowed a single spline with well-defined first and second derivatives to describe the entire, multi-generation time series for a cell. As with any smoothing approach for noisy time series, potentially real, sharp transitions in the derivative across division will be lost. Still, we capture the main features in the time derivatives of these single-cell time series using a cubic smoothing spline thus providing the basis for an analysis of gene activity over time.

### Determining thresholds for activation of transcription

For precise comparisons of promoter activation kinetics in the step tests, we needed an unbiased method to assign transcription transition times. If we model the transcription rate response to a step change in phosphate as a Heaviside function, a spline fit to simulated data yields a value at the activation time that is midway between the baseline and active transcription rates. Even with cell cycle-dependent oscillations, activation would occur on average at the time transcription rate reaches the midway point to the new, time-averaged steady state (Figure S15G). The activation threshold was automatically set at 50% of the long-time (*t* > 80min after phosphate switch) transcription rate, which represents the steady-state transcription rate of the population after activation. In this way, we determine the timing of the activation feature in each transcription trace independent of promoter strength and with accuracy only dependent on biological and analytical processes which affect all experiments equivalently (see next section). Assignment of the time transcription and localization each cross their respective thresholds is diagrammed in Figure S11.

For steady-state, low expression of tetO promoters (Fig. 3, main text), inspection of single cell traces of transcription rate for CFP and YFP showed occasional pulses of active expression from the 7xtetO promoters relative to the baseline fluctuations (Figure S6). Unlike for step tests, an appropriate threshold cannot be determined simply at 50% of the pulse maximum for several reasons. First, the pulse maximum may not reflect the true steady-state transcription rate as the “on” periods are short. Second, each pulse has a different height and midpoint and weaker pulses are more susceptible to feature degradation by spline-smoothing. Third, some periods have irregular shapes and are cannot be modeled by the step change of the Heaviside function. To distinguish between “on” and “off” periods, then, we defined an absolute transcription rate threshold between real, detectable transcription and baseline noise in measurement. We expect transcription probabilities to be the same for the YFP and CFP reporters in Fig. 3 of the main text so we required the threshold to yield similar statistics for each reporter despite CFP traces having greater baseline noise. Beginning in a range of transcription rate values known to be above baseline noise (based on the early “off” times in step tests), the threshold was decreased as low as possible (3 AU/min) without significant qualitative differences arising between the transcription probabilities for the two reporters. We then confirmed this threshold was appropriate by inspecting mRNA traces after an “on” period in transcription. For strong transcription “on” periods, the following “off” period should correspond to an exponential decay in the mRNA time series reflecting pure degradation of mRNA with the measured half-life of ~17 min [16]. This is indeed the case for strong transcription “on” periods at a threshold of 3 AU/min (Figure S16), but the exponential mRNA decay after weak “on” transcription periods is both smaller and more susceptible to smoothing making it harder to detect. An absolute threshold does introduce systematic errors in transition time assignment based on “on” period height (weak periods are detected later and for less time, stronger periods are detected earlier and longer), but the transcription rate transitions are still sharp enough that assignment errors are within the ~18 min time resolution discussed below. We therefore used a threshold of 3 AU/min to designate “on” period boundaries for all transcription features in the low expression experiment.

### Time accuracy of the calculated transcription transition times

Inferring transcription rates from measured protein levels limits the time resolution of transcription events. We are particularly interested in the ability to infer large *transitions* in transcription rate and do so by applying Eqns. (1) and (2) to splines fitting total observable protein time series. There are three main sources of error which can limit our accuracy in determining these transitions. (*i*) Measurement noise at the protein level degrades feature boundaries and can obscure the times at which the true transcription rate changes. (*ii*) Cell-to-cell variability in the mRNA degradation rate used in Eqn. (2) introduces uncertainty when inferring transcription using the average mRNA degradation rate. Also, for inference we use a model where mRNA degradation rate is deterministic, but there are stochastic fluctuations in mRNA numbers during degradation. These effects should not decrease the time resolution of our inference at low expression levels by more than 17 min, the mean lifetime of a single mRNA [16]. (*iii*) Maturation of the protein fluorophore creates an observational lag in the protein time series tightly distributed around an ~10 min time-scale (Figure S12A, as discussed above). We neglect contributions from cell-to-cell variability in maturation (mostly due to errors in fitting short, noisy time series) as well as translation rate, which is relatively faster and likely also tightly distributed. We argue in the last part of this document and in Figure S17 that translation capacity with respect to our reporter genes is approximately constant over the cell cycle.

We then used simulated data to estimate the time resolution considering the three main limitations. Transcription rate and mRNA time series were generated as in Figure S15 but excluding cell cycle-dependent oscillations for clarity. Immature protein *I*(*t*) was simulated as an intermediate between mRNA and observable protein:

$$I(t)=k_{t}u(t)e^{-k_{m}t}\int e^{k_{m}t}M_{sim}(t)dt$$

where *k_m_* is the maturation rate. The observable, mature protein *P_sim_*(*t*) was then simulated similar to Eqn. including the measurement noise term:

$$P_{sim}(t)=k_{m}\int I(t)dt+\xi(t)$$

For a square pulse transcription rate time series input (Figure S18A), 1000 mature protein data series were simulated, and a transcription rate series was inferred for each by splining and using only Eqns. (1) and (2) of the Methods (not accounting for a maturation step, as in Figure S15). Transition times for activation and deactivation were defined as when the inferred transcription time series rose above or fell below the 50% threshold, respectively. Compared to the input transition times of both activation at *t* = 0 min and deactivation at *t* = 200 min, the inferred times were delayed by ~15 min on average and had a mean absolute deviation (MAD) of ~1 min (Figure S18B). Next, we allowed the mRNA degradation rate to vary during simulation of Eqn.. For each of the 1000 traces generated, the degradation rate was randomly chosen from a normal distribution of mean 0.04 min^-1^ (~17 min half-life) with a standard deviation of 0.009 min^-1^ equal to the error measured in [16]. Transcription time series were inferred from these samples using the average degradation rate, which lead to shifts in the active steady state and, thus, the appropriate threshold. Calculating transition times using the original midpoint threshold for these transcription time series does not influence the accuracy of the inferred activation time, but inferred deactivation times now have ~8.4 min MAD (Figure S18C). The transition times estimated from each inferred transcription rate time series are therefore accurate within a ~17 min window, comparable to the time-scale of mRNA degradation.

All results reported in the main text are based on inferences using only Eqns. (1) and (2), and maturation has not been accounted for in any calculation. We performed simulations combining protein maturation and measurement noise to probe the effect of maturation on inferring the transcription transition time. We find an observational lag of ~15 min, which is approximately one 5 min time point longer than the expected ~10 min delay based on the maturation half-life. As transcription is only evaluated at 5 min intervals, a threshold-crossing occurring mid-interval is treated as occurring at the next 5 min time point. The observational delay can therefore be interpreted as being 10-15 min (2-3 time points). Additionally, modeling mRNA degradation deterministically using a single, average rate for each cell limits the time accuracy to ~17 min. All observed transcription rate transition events must then be interpreted as having occurred 10-15 min in the past with a resolution of no worse than ~17 min (between 3 and 4 time points). This is a conservative estimate for activation transition times, which are not strongly influenced by mRNA degradation.

# Supplementary Discussion

## Translational capacity and cell-cycle

Increased protein production in S/G2 may be due to either increases in mRNA level or translational capacity (*k_t_* in Eqn. (1)); we argue for the former. First, while ribosomes numbers and activity are known to increase in yeast in S/G2 [17, 18], ribosome number is generally not considered rate-limiting for any particular gene as increasing gene dosage or mRNA number by transcriptional regulation leads to increased gene expression. Second, recent work in budding [19] and fission [20] yeast suggests mRNA levels of constitutive genes increase during S/G2. Third, we find average protein to mRNA ratios of cells grouped by cell-cycle phase to show no discernible cell-cycle dependent trend (Figure S17).

## Stochastic models of cell-cycle dependent transcription for P_1/7xtetO_ and P*_DOA1_*

Standard stochastic models of gene expression describe promoter fluctuations between two states, ON and OFF, with exponential waiting times in both states. Conditions of transcriptional bursting occur when the promoter rarely transitions to the ON state (with some burst frequency) and spends a short but productive period producing mRNA (with some burst size). This process is predicted to yield a negative binomial distribution of mRNA at stationary conditions [21]. Our measured mRNA distributions *are* well-fit by this distribution (Fig. 4, black) and the inferred burst sizes range from 5-8 mRNA for P_1xtetO_ and 9-10 for P_7xtetO_ mRNA across basal and intermediate expression levels. P*_DOA1_* expression leads to nearly Poisson-like distributions, with a burst size only slightly greater than 1 mRNA (Supplementary Table 3). These burst parameters are consistent with previous measurement [16, 22]. However, our observations that mRNA profiles vary throughout the cell cycle clearly contradict these current models.

We modified standard stochastic models for gene expression to incorporate cell-cycle effects and see if these could describe observed mRNA distributions. The model uses different but fixed transcription rates in G1 (*k_txn_/f*) and S/G2/M (*k_txn_*):

$$\frac{dM}{dt}=\left\{\begin{matrix} k_{txn}/f-\gamma_{M}M & 0<t\leq t_{G1} \\ k_{txn}-\gamma_{M}M & t_{G1}<t\leq t_{C} \end{matrix}\right\}$$

with *f* defined as the ratio of transcription in S/G2/M to G1. Model parameters set by experimental observations include: a 20 min vYFP mRNA half-life (*ln(2)/γ_M_*) [16] a 120 min cell cycle duration (*t_C_*), and a 55 min G1 duration (*t_G1_*), with the latter two parameter values varying slightly depending on the sample. We use a finite state Markov approach [23] to simulate the stochastic birth and death of mRNA across the cell cycle. This yields the mRNA distribution as a function of cell-cycle progression. The stationary condition is enforced such that the beginning mRNA distribution is the result of binomial partitioning of the end mRNA distribution. For example, for *f = 1*, this model is well-known to yield a stationary mRNA distribution that is Poisson with mean $k_{txn}/\gamma_{M}$ if one explicitly ignores cell-cycle. Our solution is an oscillatory steady-state because the initial mRNA distribution in new cells right after cytokinesis is consistent with the binomial partitioning of mRNA present in the mother plus bud right before cytokinesis. While much is known about the age-dependent structure and size distribution of yeast populations, we do not attempt to describe these details in this or other models. These details do not have large qualitative effects and omitting them provides simplicity without sacrificing our ability to assess the importance of cell-cycle dependent transcription.

The choice of the two remaining unspecified parameters, *k_txn_* and *f*, dictates the relative importance of txn in G1 vs S/G2/M. We evaluated 3 different choices: setting *f*=1 such that *k_txn_* is constant throughout the cell cycle, setting *f*=2 consistent with the expected increased due to gene dosage, and allowing *f>2*. With the first two choices, *f* is set and *k_txn_* is specified such that the mean of the measured and model distributions are equivalent. For the last choice, *f* and *k_txn_* are specified such that the mean of the measured and model G1 and S/G2/M distributions match. This always leads to *f* > 2. Because each choice corresponds to a different way of modeling cell-cycle dependent transcription, we will refer these as separate models. We evaluated the relative performance of each model both qualitatively by visual comparison of the progression of model and experimental distributions over the cell cycle (Fig. 4, Figure S8) and in G2 specifically (Figure S7); and quantitatively, by evaluating a χ^2^ goodness of fit (Supplementary Table 3) and comparing the ratio of the mean mRNA number in G1 to the mean mRNA number at the end of the cell cycle, defined as $R_{M}=\left\langle M(t_{c})\right\rangle/\left\langle\int_{t=0}^{t=t_{G1}} M(t)\right\rangle$ (Supplementary Table 3). The latter quantitative metric is estimated experimentally ($\hat{R}_{M}$) as the ratio of the mean mRNA in cells with the largest bud size (late G2/M) with cells in G1, and hence will be biased downward. This is a slightly different way of examining the models because it ignores the early/mid S/G2 data, but it gets directly to whether a particular model is capable of describing the relative (S/G2/M versus G1) increase in mRNA number across the cell-cycle.

Not surprisingly, the *f=1* model clearly underpredicts the difference between mRNA levels in G1 and S/G2/M (Figure S7, black) for all sets of experimental data. This is also reflected in the quantitative metrics (Supplementary Table 3), with a model prediction of *R_M_* = 1.2, where experimental estimates of$\hat{R}_{M}$ are much larger than 1.2. In contrast, the *f=2* model is successful at fitting the P*_DOA1_* total mRNA distributions, both qualitatively and quantitatively (Fig. 4 green, Supplementary Table 3). However the S/G2/M mRNA distributions are better fit by a model with *f*>2 (Figure S7, red) and the experimentally observed $\hat{R}_{M}$ of 2.4 and 4.2 correspond to *f* values of 2.7 and 8.9 (Supplementary Table 3). Taken together, the data appears consistent with relative S/G2/M to G1 transcription rates of 2-fold or more. When *f* and *k_txn_* are simultaneously varied to match the G1 and S/G2/M means, the tetO promoter distributions are better predicted qualitatively and quantitatively (Fig. 4; Figure S7, red, Supplementary Table 3), with *f* > 2 reflecting an increase in G2 transcription rate beyond gene dosage. This is supported by experimental $\hat{R}_{M}$ values corresponding to *f* values >3 (Supplementary Table 3).

Still, none of the models successfully describe the large variance in the mRNA distribution seen especially for P_7xtetO_. Because of the variability we observed in when cells transition to the G2 transcription rate (Fig. 3B), we introduced this into our model mandating the transition between G1 and S/G2/M transcription rates as occurring in a uniformly distributed 40 minute window starting at the beginning of S/G2/M. This variable timing model results in distributions (Figure S8, orange) better able to describe the observed variance. For both basal and intermediate expression levels from P_1xtetO_ and intermediate expression from P_7xtetO_, the model passes the χ^2^ goodness of fit test (Supplementary Table 3). The measured P_7xtetO_ expression is still more variable than this model. These results in no way exclude the possibility of other sources of variability – for example adding transcriptional bursting during S/G2/M can also describe the variability, including the increased variability in P_7xtetO_ (also see below). But taken together, the variability in the observed mRNA distributions can be explained by the dependence of transcription rate on cell-cycle phase for P*_DOA1_*, a low noise, constitutive, housekeeping gene, but requires an additional source of variability for the noisy tetO promoters.

## Intrinsic noise in tetO promoter expression

Some portion of the variability is still due to “intrinsic” fluctuations – such as bursts of expression. The 3-color diploid results (Fig. 3) demonstrate that the actual transcriptional activity / protein production of two identical promoters in the productive S/G2/M phase can be *very* different. When one promoter turns on and the other does not in S/G2/M, this must be due to some intrinsic fluctuation in *cis*. Moreover, we did a two-color FISH experiment on a diploid yeast cell expressing YFP (Venus) and RFP (tdTomato) on homologous copies of P_1xtetO_. Although for technical reasons we had difficulty reliably counting individual spots of RFP mRNA, we had no trouble visualizing the bright nascent spots (Figure S9). The fraction of RFP and YFP nascent spots was nearly equivalent and strongly correlated: for basal expression (N=124) there were 32±6% and 39±7% of cells with RFP and YFP nascent spots respectively and *ρ* = 0.64; for intermediate expression (N=143) there were 30±6% and 31±6% of cells with RFP and YFP spots, respectively and *ρ* = 0.61. A partial correlation can be expected just by restricting expression to a short portion of the cell cycle, even if the appearance of the spots is independent within that portion. A more rigorous analysis requires characterization of the mean lifetimes of the two nascent spots, which may be different because of the difference in tdTomato and Venus transcript length. Nevertheless, the partial correlation suggests some degree of intrinsic fluctuations which is consistent with the real-time data. Finally, the appearance of bright nascent spots suggests “bursty” expression. Nevertheless, while both the standard “transcriptional bursting” model and the cell-cycle dependent transcription models suggested here are capable of resulting in mRNA distributions with negative binomial statistics, only the latter are consistent with our experimental observations.

# Supplementary References

1. Wykoff DD, Rizvi AH, Raser JM, Margolin B, O'Shea EK. (2007) Positive feedback regulates switching of phosphate transporters in S. cerevisiae. Mol Cell 27: 1005-1013.

2. Bun-Ya M, Nishimura M, Harashima S, Oshima Y. (1991) The PHO84 gene of saccharomyces cerevisiae encodes an inorganic phosphate transporter. Mol Cell Biol 11: 3229-3238.

3. Thomas MR, O'Shea EK. (2005) An intracellular phosphate buffer filters transient fluctuations in extracellular phosphate levels. Proc Natl Acad Sci U S A 102: 9565-9570.

4. Wykoff DD, O'Shea EK. (2001) Phosphate transport and sensing in saccharomyces cerevisiae. Genetics 159: 1491-1499.

5. Ogawa N, Oshima Y. (1990) Functional domains of a positive regulatory protein, PHO4, for transcriptional control of the phosphatase regulon in saccharomyces cerevisiae. Mol Cell Biol 10: 2224-2236.

6. McAndrew PC, Svaren J, Martin SR, Horz W, Goding CR. (1998) Requirements for chromatin modulation and transcription activation by the Pho4 acidic activation domain. Mol Cell Biol 18: 5818-5827.

7. Griesbeck O, Baird GS, Campbell RE, Zacharias DA, Tsien RY. (2001) Reducing the environmental sensitivity of yellow fluorescent protein. mechanism and applications. J Biol Chem 276: 29188-29194.

8. Sheff MA, Thorn KS. (2004) Optimized cassettes for fluorescent protein tagging in saccharomyces cerevisiae. Yeast 21: 661-670.

9. Hirst K, Fisher F, McAndrew PC, Goding CR. (1994) The transcription factor, the cdk, its cyclin and their regulator: Directing the transcriptional response to a nutritional signal. EMBO J 13: 5410-5420.

10. Gordon A, Colman-Lerner A, Chin TE, Benjamin KR, Yu RC, et al. (2007) Single-cell quantification of molecules and rates using open-source microscope-based cytometry. Nat Methods 4: 175-181.

11. Di Talia S, Skotheim JM, Bean JM, Siggia ED, Cross FR. (2007) The effects of molecular noise and size control on variability in the budding yeast cell cycle. Nature 448: 947-951.

12. Cookson NA, Cookson SW, Tsimring LS, Hasty J. (2010) Cell cycle-dependent variations in protein concentration. Nucleic Acids Res 38: 2676-2681.

13. Cross FR. (1995) Starting the cell cycle: What's the point? Curr Opin Cell Biol 7: 790-797.

14. Savitzky A, Golay MJE. (1964) Smoothing and differentiation of data by simplified least squares procedures. Anal Chem 36: 1627-1639.

15. De Boor C. (2001) A practical guide to splines. New York: Springer-Verlag. 346 p.

16. To TL, Maheshri N. (2010) Noise can induce bimodality in positive transcriptional feedback loops without bistability. Science 327: 1142-1145.

17. Waldron C, Jund R, Lacroute F. (1977) Evidence for a high proportion of inactive ribosomes in slow-growing yeast cells. Biochem J 168: 409-415.

18. Elliott SG, McLaughlin CS. (1978) Rate of macromolecular synthesis through the cell cycle of the yeast saccharomyces cerevisiae. Proc Natl Acad Sci U S A 75: 4384-4388.

19. Trcek T, Larson DR, Moldon A, Query CC, Singer RH. (2011) Single-molecule mRNA decay measurements reveal promoter- regulated mRNA stability in yeast. Cell 147: 1484-1497.

20. Zhurinsky J, Leonhard K, Watt S, Marguerat S, Bahler J, et al. (2010) A coordinated global control over cellular transcription. Curr Biol 20: 2010-2015.

21. Paulsson J, Berg OG, Ehrenberg M. (2000) Stochastic focusing: Fluctuation-enhanced sensitivity of intracellular regulation. Proceedings of the National Academy of Sciences of the United States of America 97: 7148-7153.

22. Zenklusen D, Larson DR, Singer RH. (2008) Single-RNA counting reveals alternative modes of gene expression in yeast. Nat Struct Mol Biol 15: 1263-1271.

23. Munsky B, Khammash M. (2006) The finite state projection algorithm for the solution of the chemical master equation. J Chem Phys 124: 044104.

24. Boehlke KW, Friesen JD. (1975) Cellular content of ribonucleic acid and protein in saccharomyces cerevisiae as a function of exponential growth rate: Calculation of the apparent peptide chain elongation rate. J Bacteriol 121: 429-433.

25. von der Haar T. (2008) A quantitative estimation of the global translational activity in logarithmically growing yeast cells. BMC Syst Biol 2: 87.

26. Raser JM, O'Shea EK. (2004) Control of stochasticity in eukaryotic gene expression. Science 304: 1811-1814. 10.1126/science.1098641.

27. Lee TH, Maheshri N. (2012) A regulatory role for repeated decoy transcription factor binding sites in target gene expression. Mol Syst Biol 8: 576.

28. Longtine MS, McKenzie A,3rd, Demarini DJ, Shah NG, Wach A, et al. (1998) Additional modules for versatile and economical PCR-based gene deletion and modification in saccharomyces cerevisiae. Yeast 14: 953-961.

29. Nevoigt E, Kohnke J, Fischer CR, Alper H, Stahl U, et al. (2006) Engineering of promoter replacement cassettes for fine-tuning of gene expression in saccharomyces cerevisiae. Appl Environ Microbiol 72: 5266-5273.
